# Energy Transport in Dichroic Metallo‐organic Crystals: Selective Inclusion of Spatially Resolved Arrays of Donor and Acceptor Dyes in Different Nanochannels**

**DOI:** 10.1002/anie.202214041

**Published:** 2022-12-16

**Authors:** Qiang Wen, Naveen Malik, Yoseph Addadi, Maren Weißenfels, Vivek Singh, Linda J. W. Shimon, Michal Lahav, Milko E. van der Boom

**Affiliations:** ^1^ Department of Molecular Chemistry and Materials Science Weizmann Institute of Science Rehovot 7610001 Israel; ^2^ Department of Life Science Core Facilities Weizmann Institute of Science Rehovot 7610001 Israel; ^3^ Department of Chemical Research Support Weizmann Institute of Science Rehovot 7610001 Israel

**Keywords:** Dichroic Crystals, Förster Resonance Energy Transfer, Metal–Organic Frameworks, Selective Guest Inclusion

## Abstract

In this study, the precise positioning and alignment of arrays of two different guest molecules in a crystalline host matrix has been engineered and resulted in new optically active materials. Sub‐nm differences in the diameters of two types of 1D channels are sufficient for size‐selective inclusion of dyes. Energy transport occurs between the arrays of different dyes that are included in parallel‐positioned nanochannels by Förster resonance energy transfer (FRET). The color of individual micro‐sized crystals are dependent on their relative position under polarized light. This angular‐dependent behavior is a result of the geometrically constrained orientation of the dyes by the crystallographic packing of the host matrix and is concentration dependent.

## Graphical abstract

Förster resonance energy transfer (FRET) between arrays of donor‐acceptor chromophores in a metal–organic framework has been observed. These arrays of aligned chromophores have been formed by size‐selective inclusion in differently nanosized channels, resulting in micro‐sized crystals with anisotropic optical properties.

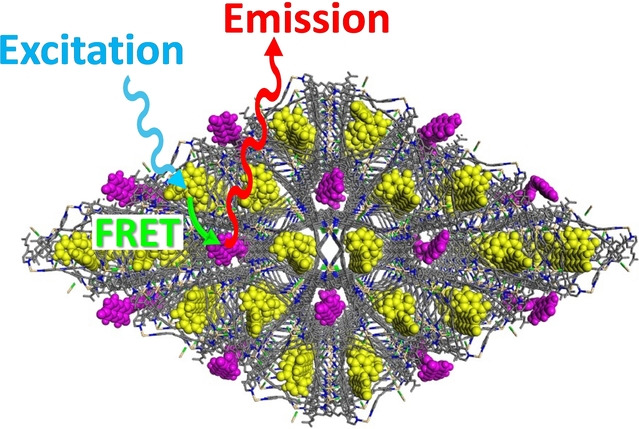

## Introduction

The concept and the development of molecular host–guest chemistry have resulted in many fascinating and emerging technologies.^[1]^ The rules of this branch of supramolecular chemistry have been applied to a large diversity of materials.^[2]^ Much work in this field has been inspired by the seminal work of Bayer and Wilchek on the avidin‐biotin pair that has become a general tool in the biological sciences.^[3]^ An example is the use of the complementarity of DNA strands to assemble metallic nanoparticles in larger structures as diagnostic tools by Mirkin.^[4]^ Chromophores stability and also that of highly unstable compounds such as white phosphorus can be enhanced by selective encapsulation into metal–organic cages.[^5^, ^6^] The sieving properties of metal–organic frameworks (MOFs) have been exploited for energy storage, carbon capture and membrane technology.[^7^, ^8^, ^9^] Inclusion of chromophores in these porous crystals opens up many new possibilities for the generation of new optically functionalized materials by post‐assembly.[^10^, ^11^, ^12^, ^13^, ^14^, ^15^, ^16^, ^17^, ^18^] The development of such materials is not trivial. Among many factors, pore size, shape and geometry should obviously be correlated with the nature of the guest molecules. For example, Harris, Guillaume et al. reported organic crystals with multicomponent inclusion and controlled guest arrangements during the growth process.^[19]^ Guest molecules were also placed in different areas and shells of MOFs.[^13^, ^14^, ^20^] A major challenge is the exact positioning and alignment of different chromophores in a crystalline host matrix.^[17]^ Optically functional crystals have been used to demonstrate and study Förster resonance energy transfer (FRET) by Hupp,^[21]^ Farha,^[22]^ Lin,^[23]^ Morris^[24]^ and others.[^25^, ^26^, ^27^, ^28^] For example, FRET has been reported between the MOF skeleton to an embedded coumarin dye.^[23]^ White light emission has been demonstrated by including mixtures of dyes in MOFs by post‐assembly, and by encapsulation of dyes into pores during crystal formation resulting in functional multishell structures.[^14^, ^26^, ^28^] A number of groups reported FRET using dye molecules in covalent organic frameworks (COFs).[^29^, ^30^, ^31^]

Other factors hampering the development of such materials are the unpredictability and limited correlation between crystallographic structures and crystal morphologies. Moreover, low uniformity and lack of monodispersity limit the further use of individual single‐crystals. The work by Al‐Ghoul and Hmadeh is an example of making differently sized and uniform MOFs.^[32]^

**Scheme 1:**
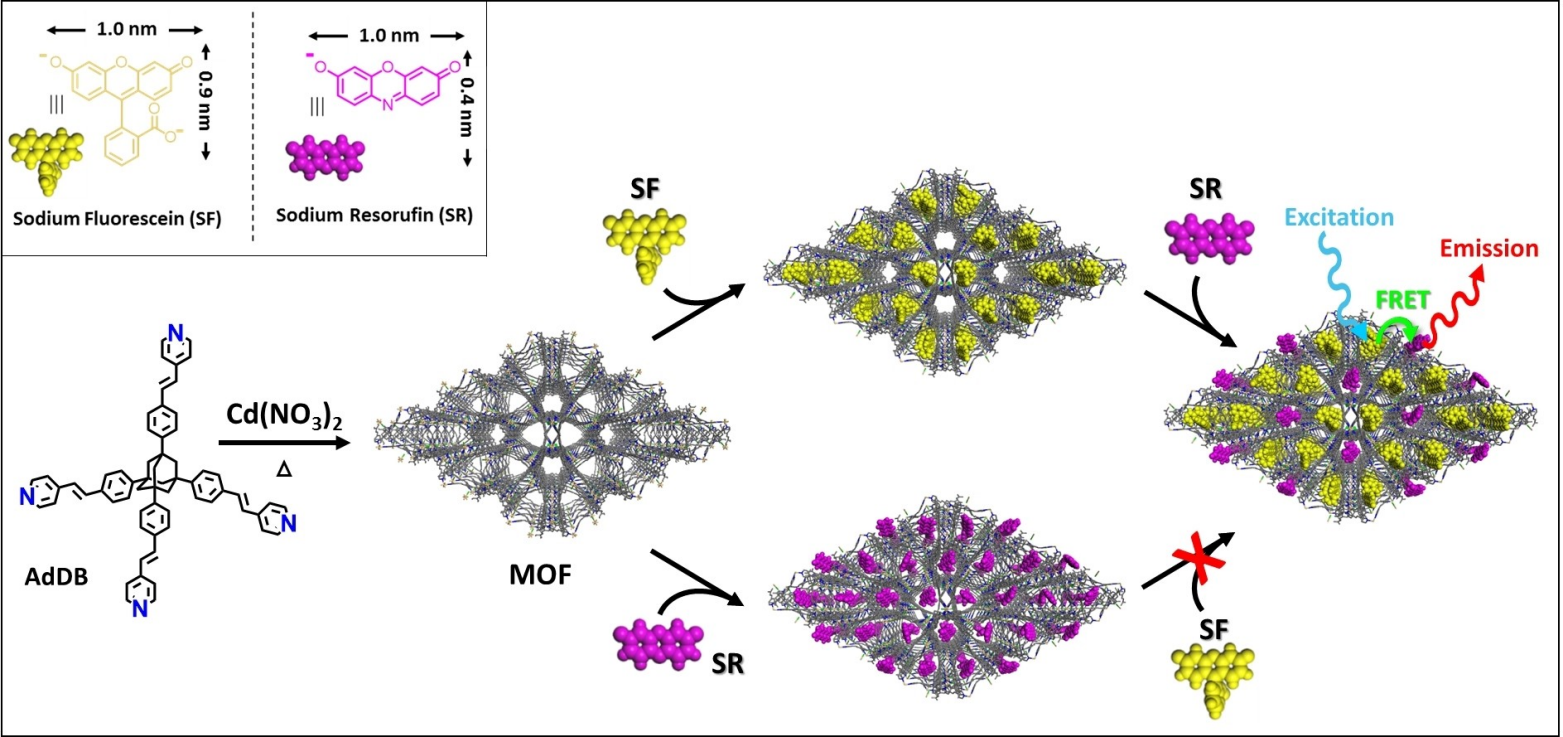
The solvothermal reaction of an organic ligand with cadmium nitrate in dimethylformamide at 105 °C for 24 hours results in the formation of colorless and uniformly shaped crystals. These crystals have two differently sized nanochannels with diameters of about 1.0 nm and 0.7 nm. The parallel and continuous channels can be selectively functionalized with pairs of donor‐acceptor chromophores, resulting in dichroic crystals that are suitable for light‐conversion applications.

We have demonstrated recently that the organic ligand, **AdDB**,^[33]^ shown in Scheme 1 and similar systems form isostructural crystals with first row, bivalent metals (i.e., manganese, iron, cobalt, nickel, copper and zinc).[^34^, ^35^, ^36^, ^37^, ^38^] The crystals have two types of continuous and chiral channels aligned along the crystallographic *c*‐axis. This structural feature is atypical,[^39^, ^40^, ^41^] and has great potential for post‐functionalization by size‐selectively inclusion of other components as demonstrated in this study. The abovementioned bivalent metal cations favor an octahedral geometry with four pyridine ligands coordinated in the same plane, making it possible to predict the crystallographic structure for other metals favoring a similar coordination geometry. Although the use of this series of metal cations afforded a diversity of different morphologies, for most cases the crystal uniformity is high.

We demonstrate here the size‐selective inclusion of two types of dyes in different nanosized‐channels. The resulting crystals are dichroic: they exhibit color‐to‐color transitions and different color intensities when exposed to polarized light. The arrays of donor and acceptor dyes assembled in parallel channels display energy transfer by nonradiative dipole‐dipole coupling. The FRET is confirmed by fluorescence lifetime measurements in combination with photobleaching of the acceptor dye.[^42^, ^48^, ^49^] Colorless cadmium‐based crystals with the ligand, **AdDB**, were prepared as host matrix for the assembly of new materials for optical applications. Coordination of this ligand to bivalent cadmium cations resulted in the same coordination structure as recently found for other first row metals,[^34^, ^35^, ^36^, ^37^, ^38^] but these new crystals are highly transparent (similar to manganese‐based crystals).^[38]^ Previous studies have shown that the uniformity in the size and shape of isostructural crystals is higher than that of crystals formed at room temperature using the slow diffusion of one solvent into another.^[38]^ Therefore, solvothermal reaction conditions were used here to generate thermodynamically favored, stable structures that are suitable for post‐assembly functionalization.

## Results and Discussion

The new crystals are prepared by the reaction of the ligand (**AdDB**), and cadmium nitrate in dimethylformamide (DMF) under solvothermal conditions (105 °C) for 24 hours (Scheme 1). The used molar ratio of ligand‐to‐metal salt is 1 : 4.6. An acid (HCl) was added to grow micrometer‐sized, colorless crystals. The acid can slow down the crystallization process by protonation of the pyridine moieties, hence hampering the rapid coordination of the ligand to the metal centers. The crystals were collected in high yield (89 % based on the ligand) by vacuum filtration and subsequently washed with methanol.

**Figure 1:**
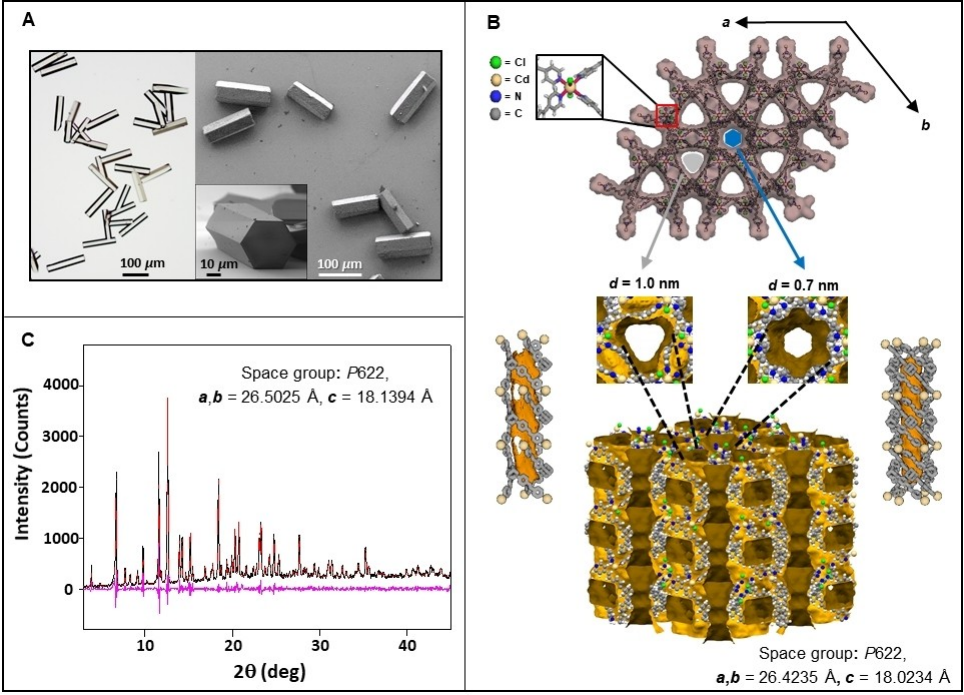
Structural characteristics of the host crystals: morphology and molecular packing. A) Optical and scanning electron microscope (SEM) images. B) Single‐crystal X‐ray data: (top) space‐filling representation viewed from the *c*‐axis with two differently nanosized channels denoted in grey (1.0 nm) and blue (0.7 nm). Zoom in: coordination node. (bottom) side perspective view showing the Connelly surface of the 1D channels and voids (brown). Zoom in: top perspective view of the chiral channels (Table S1).^[43]^ C) Experimental powder X‐ray diffraction (PXRD) spectrum (black), fitted spectrum using the single‐crystal X‐ray data (red), and the difference between the experimental and fitted spectra (pink).

Both optical and scanning electron microscope (SEM) imaging showed the formation of well‐defined hexagonal prisms having a high monodispersity (length=112±10 *μ*m for 55 crystals) (Figure 1A). Single crystal X‐ray diffraction showed a structure belonging to the chiral hexagonal 622 crystal system with the rare space group *P*622 (Figure 1B). Hexagonal plates were also observed as a minor product (<2 %) (Figure S1). Single‐crystal X‐ray diffraction studies revealed that the prisms and the plates have the same crystallographic structure (Figure S2, Table S1).^[43]^ The phase purity was confirmed by fitting the measured powder X‐ray diffraction (PXRD) diffraction patterns of bulk samples with one simulated from the crystal structure (Figure 1C). These crystals are part of our series of isostructural, chiral metal–organic frameworks grown from achiral components.[^34^, ^35^, ^36^, ^37^, ^38^] The *propeller‐type* arrangement of *four* coordinated pyridine units from *four* different ligands around the bivalent cadmium cations imparts a chiral arrangement on both the coordination nodes and the channels. Also in these new crystals, two types of channels are positioned in parallel along the *c*‐axis,[^39^, ^40^, ^41^] one hexagonal‐type centered around the 6‐fold axes and the trigonal‐type centered around the crystallographic 3‐fold axis. The inner walls of the two types of channels are of the same chirality. The distance between the centers of the two different channels is ≈1.5 nm. This distance is sufficiently close to allow energy transfer via nonradiative dipole–dipole coupling between arrays of different dyes included in these channels, as shown in this study. The trigonal and hexagonal one‐dimensional channels have diameters of 1.0 nm and 0.7 nm, respectively. The larger channels have the aromatic rings of the ligand positioned in parallel to the channel's orientation, opening up the possibility of π‐π interactions with guest molecules. The diameters of the two channels are sufficiently large to allow small dyes such as sodium resorufin to enter, as recently shown by us for isostructural crystals.^[37]^ We hypothesized here that although the difference in the diameters of the two channels is only ≈0.3 nm, it could be possible to selectively functionalize these channels with different dyes based on their dimensions. Relatively small dyes would enter both channels, whereas the larger dyes can only enter the trigonal channels.

**Figure 2:**
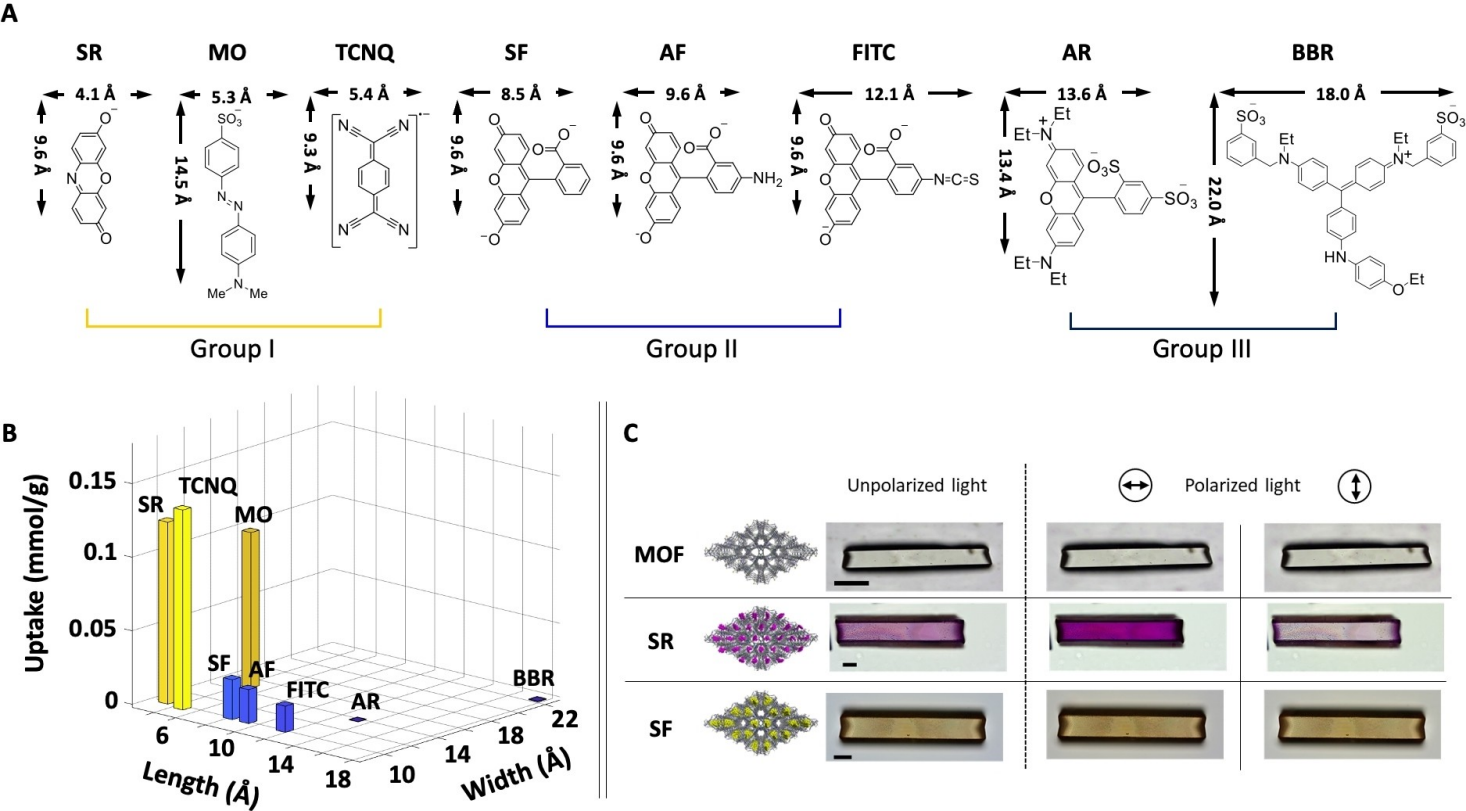
Size‐selective dye inclusion into the host crystals. A) Molecular structures and approximate dimensions (the cations are not drawn). The following dyes are sodium salts of resorufin (**SR**), methyl orange (**MO**), fluorescein (**SF**), 5‐aminofluorescein (**AF**), fluorescein isothiocyanate isomer I (**FITC**), sulforhodamine B (also known as acid red 52, **AR**), and brilliant blue R250 (**BBR**). The lithium salt of 7,7,8,8‐tetracyanoquinodimethane (**TCNQ**) was used. B) The process of molecular sieving by the colorless host crystals was monitored by UV/Vis spectroscopy of the dye suspension in methanol. The 3D plot shows the amount of dye uptake by the crystals with respect to the dyes’ length and width (Figures S3,S4; Table S2). C) Unpolarized and polarized optical microscopy images of the colorless host crystals (top), the crystals functionalized with the sodium salts of resorufin (**SR**=2.5×10^−3^ mmol g^−1^, purple, middle) or fluorescein (**SF**=0.03 mmol g^−1^, yellow, bottom). Scale bar: 30 *μ*m.

To explore and demonstrate the selective sieving effects of our crystals, we suspended them in a series of eight methanol solutions with differently‐sized dyes (Figure 2A, Figure S3). Follow‐up optical absorption (UV/Vis) measurements of the dye solutions show the gradual decrease of their concentrations for relatively small molecules. The sieving process is completed in ≈24 h. The dye loading is the highest for the three molecules that have a “width” smaller than 0.7 nm: sodium resorufin (**SR**), methyl orange (**MO**) and lithium 7,7,8,8‐tetracyanoquinodimethane (**TCNQ**) (=group I). Significantly lower amounts of dyes are embedded into the crystals when larger dyes are used with a “width” between 0.7–1.2 nm: sodium fluorescein (**SF**), sodium 5‐aminofluorescein (**AF**) and fluorescein isothiocyanate isomer I (**FITC**) (=group II). The largest molecules we used do not enter the host matrix: sodium acid red‐52 (**AR**) and brilliant blue R250 (**BBR**); width >1.2 nm (=group III) (Figure 2B). The estimated differences between the amounts of dyes embedded into the crystals for these three groups of crystals (I: 0.11–0.14 mmol g^−1^, II: 0.02–0.03 mmol g^−1^ and III: none) is a strong indication that selective functionalization of the different channels of the crystals is possible based on the dimensions of the guest molecules (Figure S4, S5, Table S2). These sieving experiments show that the smallest channel is only accessible to the dyes of group I, whereas the larger channel is accessible to both small and large dyes (groups I and II).

Optical images of isolated crystals clearly show the colors associated with the homogeneously distributed dyes. Polarized light measurements show that some of these different dyes are aligned inside the channels (Figure S4), and this effect is dependent on the concentration of the dyes. Optical images of crystals saturated with dyes recorded at different angular directions show moderate or minor color changes for the small and medium‐sized molecules (Group I and II) (Figure S4). Interestingly, crystals loaded with lower amounts of one of the smaller dyes i.e., sodium resorufin, do show clear changes in the intensity of the purple color (Figure 2C). We postulate that this change in coloration intensity is a result of different organization of sodium resorufin in the two different channels. The smaller channel is more likely to restrict the number of possible orientations resulting in an aligned array of molecules whose absorption is dependent on the incident angle of the polarized light. In contrast, the same dyes inside the larger channels are less ordered as their absorption is less affected by polarized light. Therefore, the crystals do not become completely transparent.

Experiments performed with a medium‐sized dye, sodium fluorescein (yellow, Figure 2C) do not show significant concentration‐dependence effects under polarized light. The asymmetric molecular structure of sodium fluorescein and structurally similar dyes may allow absorption of polarized light under different incident angles even when their supramolecular structure consists of arrays of aligned dyes.

**Figure 3:**
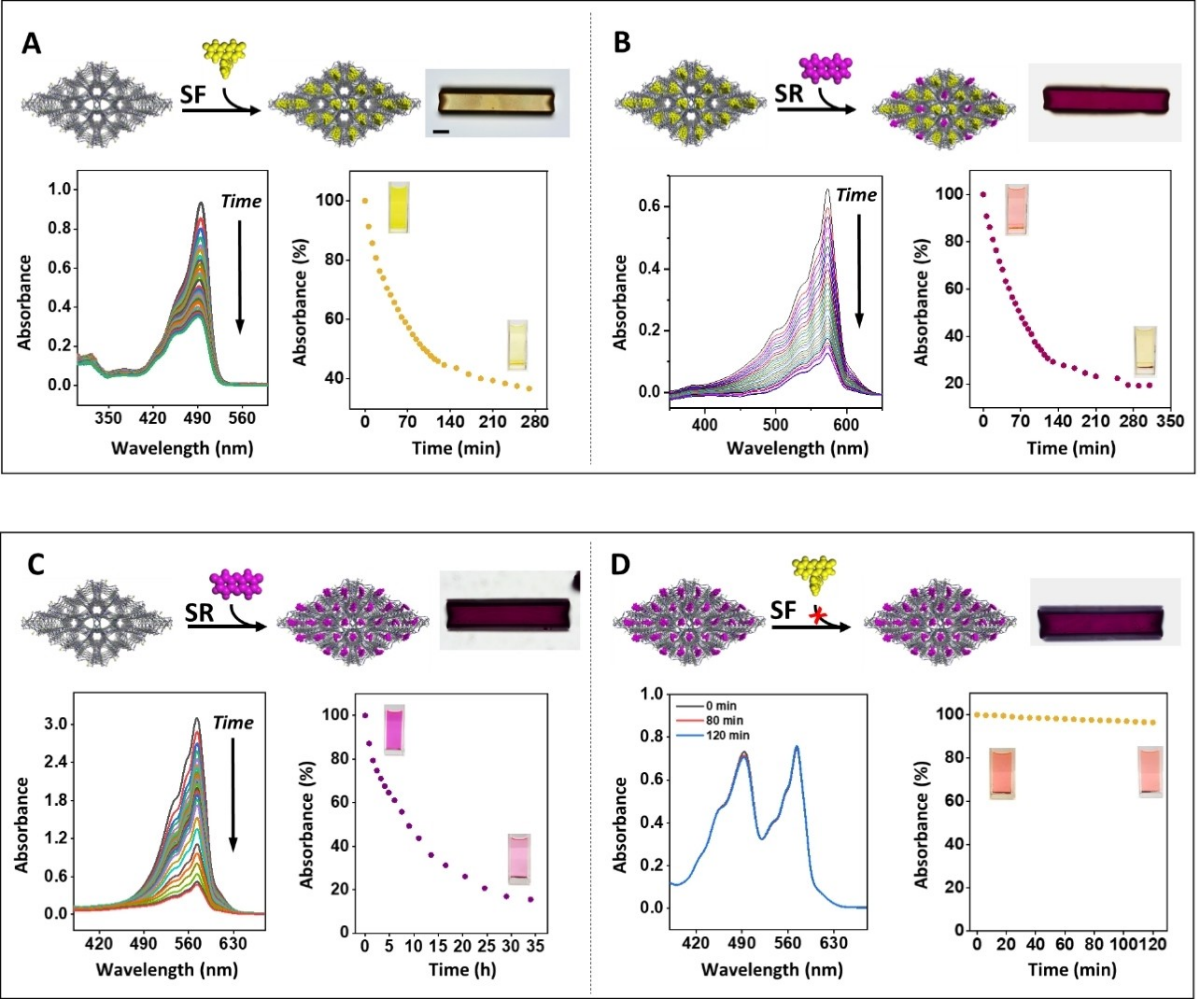
Sequence‐dependent and size‐selective functionalization of the different nanosized channels of the host crystals with the sodium salts of fluorescein (**SF**) and resorufin (**SR**). A,B) Consecutive reactions of a suspension of the colorless host crystals in methanol with **SF** and **SR**. C,D) Consecutive reactions of a suspension of the colorless host crystals in methanol with **SR** and **SF**. The optical images show the color of the crystals after the reactions with the dyes. Scale bar: 30 *μ*m. The process of molecular sieving was monitored by UV/Vis spectroscopy. The photos show the color of the suspensions in the cuvettes at the beginning and at the end of the reactions. The UV/Vis spectra shown in (A) have been used as baseline for the spectra shown in (B). The raw data are shown in Figure S6.

The demonstrated size‐selective sieving of a series of molecules is used tofunctionalize the crystals with spatially resolved arrays of different dyes. For this purpose, we selected two differently‐sized chromophores, sodium resorufin and sodium fluorescein. Such a chromophore couple can act as an energy donor‐acceptor system: by visible‐light excitation of sodium fluorescein (donor), sodium resorufin (acceptor) would emit the transferred energy.^[44]^ As described above, the crystals were first suspended in a methanol solution containing the larger dye, sodium fluorescein. This sieving process was monitored by in situ UV/Vis spectroscopy showing a gradual decrease of the concentration of sodium fluorescein in solution (Figure 3A). Optical images show the formation of yellow‐colored crystals that are saturated with the dye, filling the larger channels, while leaving the smaller channels unoccupied. Subsequent addition of the smaller sodium resorufin to the reaction solution also resulted in the absorption of the dye and purple coloration of the yellow crystals (Figure 3B, S6). The UV/Vis spectra recorded during the latter reaction do not indicate the release of sodium fluorescein from the crystals into the solution. No leaching of the two dyes was observed in methanol for 5 days by UV/Vis and fluorescence spectroscopy (Figure S7). The amount of sodium resorufin absorbed by the crystals is much less than that observed for the sieving of this dye with “empty” crystals (≈0.12 mmol g^−1^), and is in good agreement with what one would expect by functionalization of only the smaller channels (≈0.05 mmol g^−1^). To provide support for this conclusion, we first saturated the crystals with the smaller dye, sodium resorufin, and subsequently added the larger dye, sodium fluorescein, to the reaction mixture (Figure 3C,D). As can be seen from the UV/Vis data, the latter dye cannot enter the crystals saturated with sodium resorufin as the larger channels are already filled and inaccessible.

**Figure 4:**
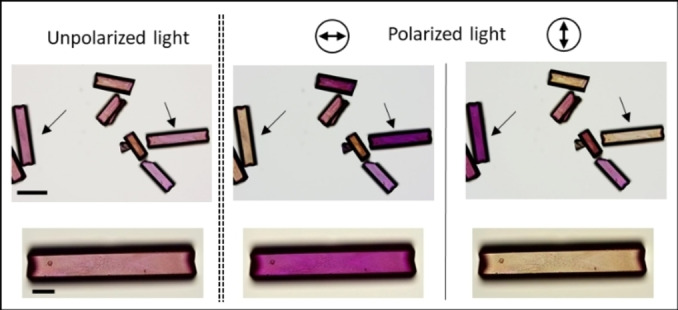
Optical microscopy images of the host crystals functionalized with both the sodium salts of fluorescein (**SF**) and resorufin (**SR**). Scale bar: 30 *μ*m. The host crystals are first saturated with **SF** (0.03 mmol g^−1^, yellow) followed by functionalization with **SR** (0.003 mmol g^−1^, purple).

The crystals functionalized with the two dyes are dichroic under polarized light (Figure 4). The sodium fluorescein‐saturated crystals were loaded with sodium resorufin for 1 hour (to have a relatively low concentration of the dye in the smaller channels) and analyzed using polarized light. Depending on the angle of the incident light in respect to the crystallographic axis, the crystals are either purple (parallel) or yellow (perpendicular). The purple color corresponds to sodium resorufin, whereas the yellow color originates from sodium fluorescein. The crystals functionalized only with sodium fluorescein do not exhibit strong polarized anisotropy, whereas crystals with sodium resorufin show clear polarization‐dependent color anisotropy at relatively low concentrations (see above, Figure 2C). The crystals are dark purple when the polarized light is oriented in parallel to the channels showing that the long axis of the dyes is confined along the crystallographic *c*‐axis. Combining these two different optical properties within a single host crystal resulted in polarization‐dependent dichroism.

Although optical dichroism is known for molecular crystals,[^39^, ^40^, ^41^] the design of such crystals using differently functionalized channels, as shown here, potentially offers a wide range of color‐to‐color transitions. We also included methyl orange (**MO**), or 7,7,8,8‐tetracyanoquinodimethane lithium salt (**TCNQ**) in crystals saturated with sodium fluorescein (**SF**) (Figures S8, S9). The scope of this self‐assembly process was further demonstrated by size‐selective functionalization of the different nanosized channels of the host crystals with the sodium salts of fluorescein isothiocyanate isomer I (**FITC**) and resorufin (**SR**) (Figure S10). The crystals functionalized with **TCNQ** and **SF**, and **FITC** and **SR** are also dichroic (e.g., green‐yellow, orange‐bordeaux).

**Figure 5:**
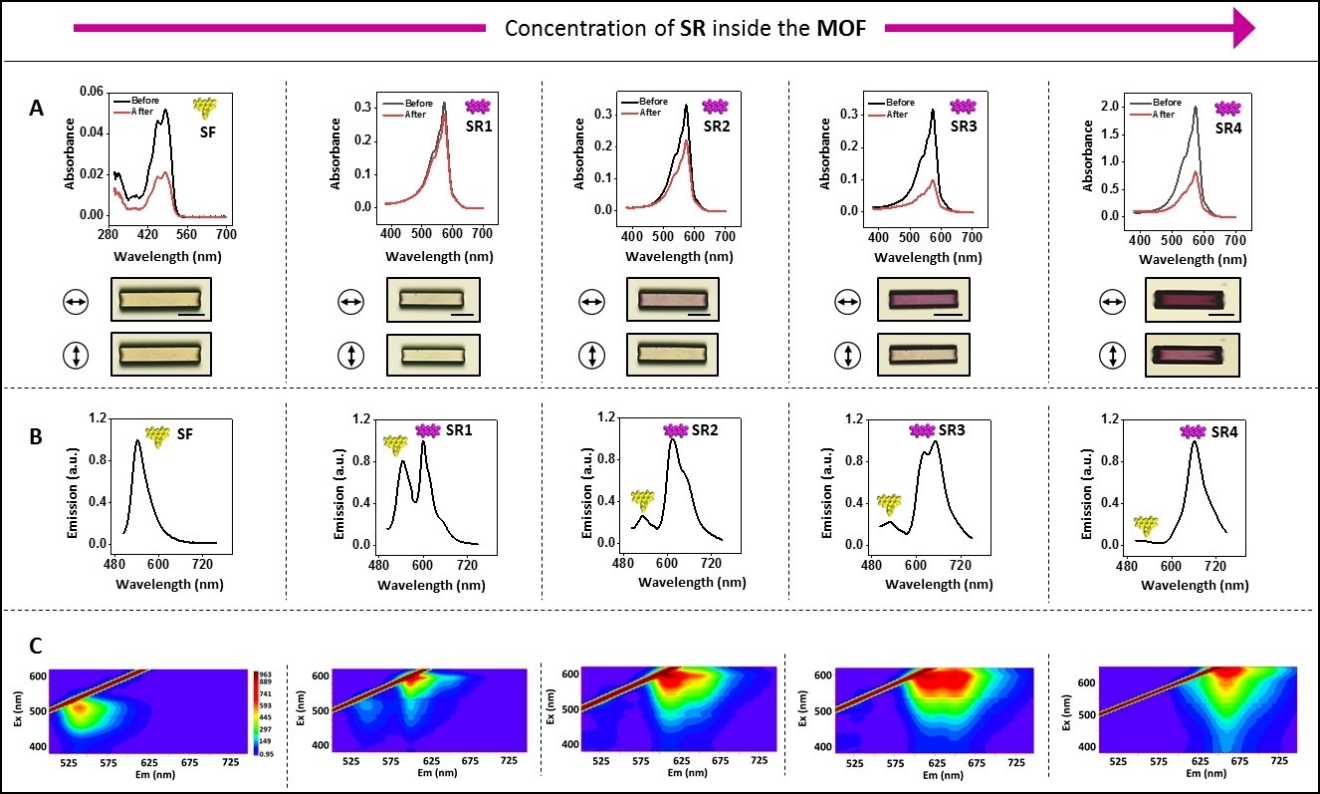
Förster resonance energy transfer (FRET) between the sodium salts of fluorescein (**SF**) and resorufin (**SR**) in the host crystals. A) UV/Vis spectra of suspensions of the host crystals in methanol with **SF** and **SR**. The host crystals were first saturated with **SF** (2.7×10^−2^ mmol g^−1^) followed by functionalization with increasing amounts of **SR** (**SR1**: 3.7×10^−5^ mmol g^−1^; **SR2**: 2.5×10^−3^ mmol g^−1^; **SR3**: 4.9×10^−3^ mmol g^−1^; **SR4**: 4.1×10^−2^ mmol g^−1^). The photos show the polarized light microscope images of single crystals with the increasing amount of **SR** (left to right). Scale bar: 50 *μ*m, B) Emission, *λ*_e*x*_=480 nm, and C) 2D fluorescence spectra of the bulk in the solid‐state with an excitation range of 375–625 nm and 5 nm intervals.

The overlap between the emission spectrum of sodium fluorescein (**SF**) and the excitation spectrum of sodium resorufin (**SR**) both in solution and included in the MOFs indicates the possibility of efficient energy transfer (Figure S11). The energy‐transfer properties and underlying mechanism of this donor‐acceptor system were studied by analyzing their fluorescence properties. The large channels of the crystals were first saturated with sodium fluorescein (=donor). Then, the smaller channels of the yellow crystals were loaded with four different amounts (**SR1**‐**SR4**) of sodium resorufin (=acceptor) by applying different soaking times. This process was monitored by UV/Vis measurements of the dye solutions resulting in purple crystals (Figure 5A). The deepening of the purple color of the dichroic crystal is visualized by polarized light microscopy. Fluorescence measurements of bulk samples show large changes upon increasing the amount of sodium resorufin (**SR1**→**SR4**) when exciting the donor at λ=480 nm (Figure 5B). Upon increasing the amount of sodium resorufin (acceptor) in the crystals gradually, the emission of the donor (λ =541 nm) becomes weaker while the emission of the acceptor (λ =608 nm) becomes stronger. A similar effect can be seen when exciting the donor in the range λ=375–625 nm (Figure 5C). The steady‐state study shows that the emission peak of donors in **SR3** and **SR4** are almost quenched. These changes are strongly indicative for an energy‐transfer process.[^23^, ^45^, ^46^, ^47^] We also observed the appearance of a new emission band at higher wavelengths (λ=648 nm), whereas the initial band of the donor becomes weaker. This effect may be indicative of different stacking of sodium resorufin at higher concentrations.

**Figure 6:**
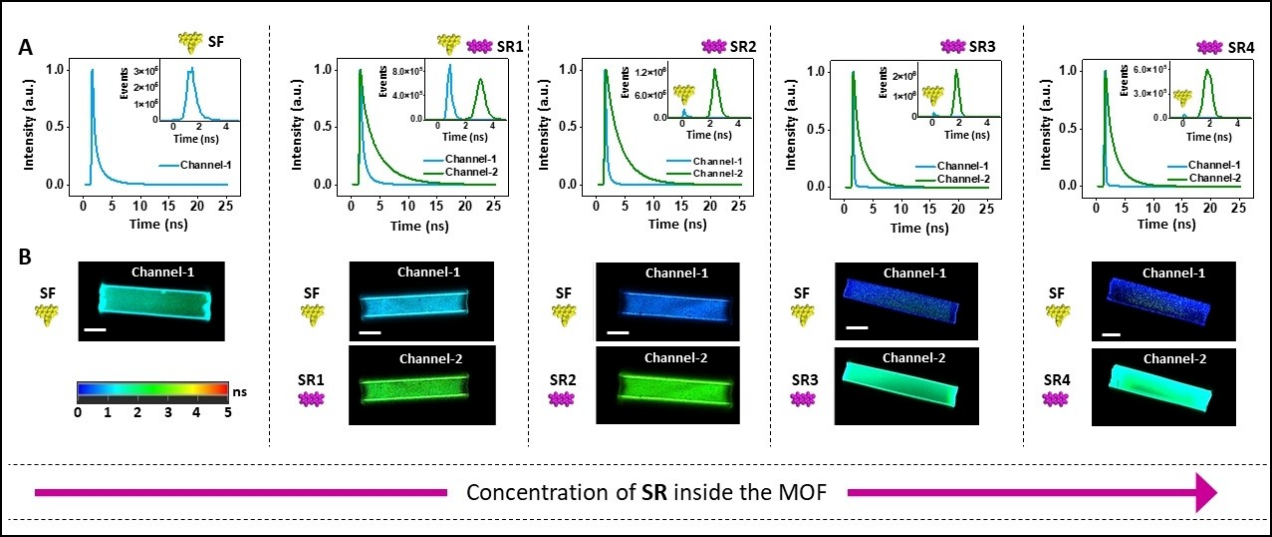
Demonstration of Förster resonance energy transfer (FRET) between the sodium salts of fluorescein (**SF**) and resorufin (**SR**) in the host crystals with confocal fluorescence lifetime imaging microscopy (FLIM). The host crystals were first saturated with **SF** (2.7×10^−2^ mmol g^−1^) followed by functionalization with increasing amounts of **SR** (**SR1**: 3.7×10^−5^ mmol g^−1^; **SR2**: 2.5×10^−3^ mmol g^−1^; **SR3**: 4.9×10^−3^ mmol g^−1^; **SR4**: 4.1×10^−2^ mmol g^−1^). A) Fluorescence lifetimes of the bulk, lifetime analysis and curve fitting is provided in the Supporting Information (Figure S12, Table S3). B) Single‐crystal fluorescence lifetime of **SF** (500–540 nm, channel‐1) and **SR** (650–700 nm, channel‐2). Scale bar=20 *μ*m. For additional fluorescence lifetime and intensity images, see Figure S13–S19.

Fluorescence measurements on the same set of individual crystals show a decrease in the lifetime for the donor as a function of the amount of the acceptor (0.702 ns to 0.08 ns, as one would expect for a FRET‐based mechanism (Figure 6A).[^23^, ^46^, ^47^] If energy transfer would solely occur via re‐absorption events, the lifetime of the donor would not be affected.^[47]^ The energy transfer efficiency, *η*_ET_=1−τ_DA_/τ_D_ (τ=lifetime),^[17]^ is becoming larger upon increasing the concentration of the acceptor (**SR1**: 23 %, **SR2**: 61 %, **SR3**: 84 %, **SR4**: 88 %) (Table S4). The lifetime of the acceptor is changing for the crystals with the highest concentration of this dye, which is in agreement with different stacking interactions.

Confocal fluorescence lifetime imaging microscopy (FLIM) showed the homogeneous distribution of the dyes and the energy transfer across the entire crystals (Figure 6B, S13, S14). The FLIM images of crystals loaded with sodium fluorescein (=donor) and sodium resorufin (=acceptor) were obtained by exciting the donor at λ=470 nm and detecting the emission at λ=500–540 nm (=emission from the donor) and at λ=650–700 nm (=emission from the acceptor). *Z*‐scanning of crystals saturated with sodium fluorescein revealed that these guest molecules are distributed across the whole crystal (Figure S15–S19). In accordance with the steady‐state emission spectrum, FLIM images of crystals saturated with sodium fluorescein and with different amounts of sodium resorufin showed also the varied degree of energy transfer between this donor—acceptor pair.

**Figure 7:**
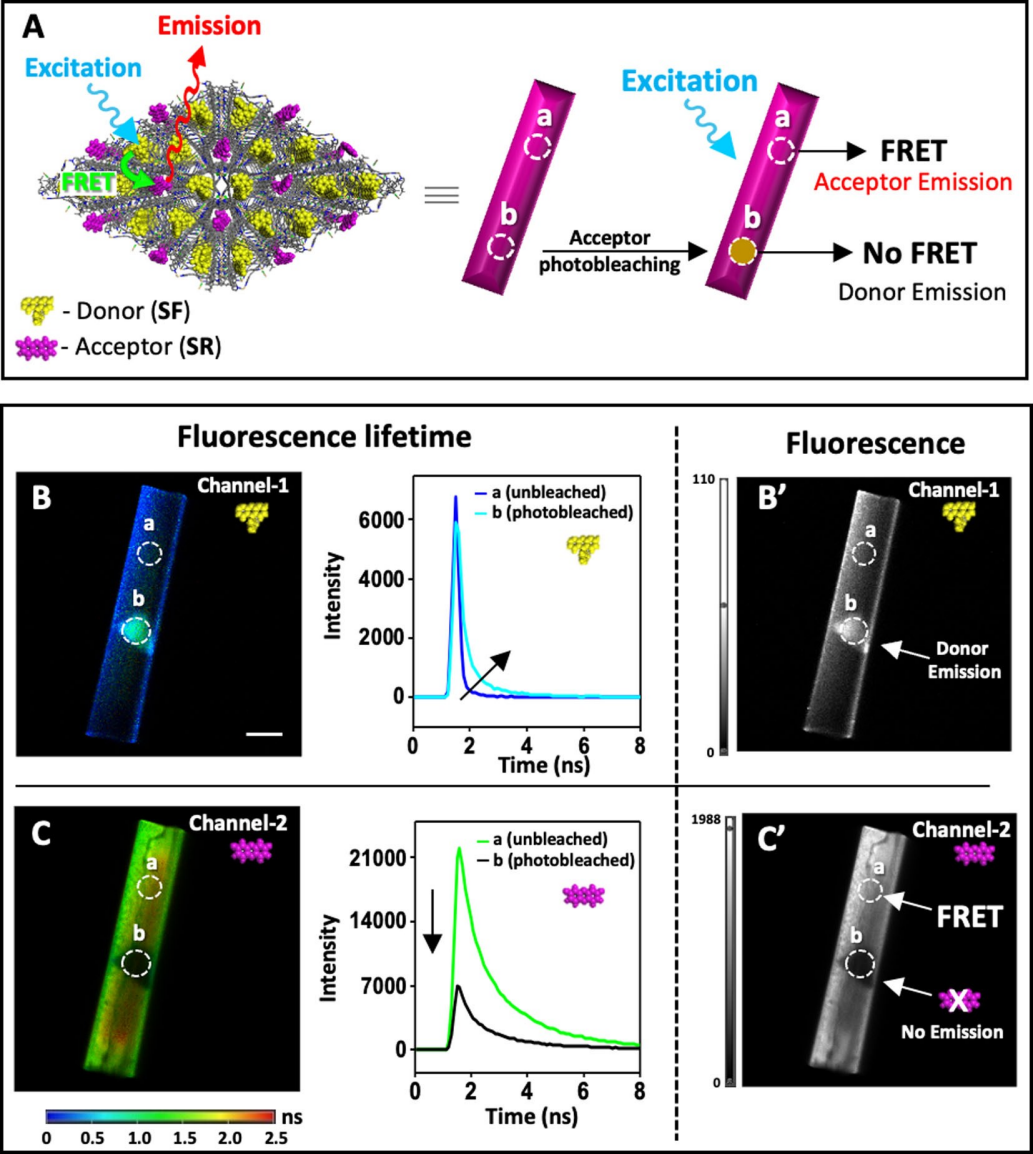
A) Schematic representation showing the Förster resonance energy transfer (FRET) of a single crystal before and after photobleaching at λ=600 nm of a selective area of a crystal functionalized with both sodium fluorescein (**SF**) and sodium resorufin (**SR**). The circles indicate unbleached (a) and photobleached areas (b). The size of the photobleached area is ca. 330 *μ*m^2^. The corresponding lifetime plots (center) are obtained by measuring areas within circles a and b. B,C) Confocal fluorescence lifetime imaging microscopy (FLIM), fluorescence lifetimes and intensity images of donor **SF** (B and B′) and acceptor dye **SR** (C and C′). Emissions of **SF** (500–540 nm, channel‐1) and **SR** (650–700 nm, channel‐2). Scale bar: 20 *μ*m. The host crystals are first saturated with **SF** (0.03 mmol g^−1^) followed by functionalization with **SR** (0.04 mmol g^−1^). Lifetime analysis and curve fitting are provided in the Supporting Information (Figure S20, Table S5).

The interaction between the different supramolecular structures is further evident by photobleaching of the acceptor (**SR**) by irradiation of small areas on individual crystals at λ=600 nm as shown schematically in Figure 7A.[^48^, ^49^] This experiment resulted in the recovery of the emission of the donor (**SF**) and its original lifetime—whereas the unexposed areas of the crystals continue to display energy transfer. Figures 7B and 7C show the fluorescence lifetimes of the donor (**SF**) and acceptor (**SR**), respectively. It can be seen that photobleaching of the acceptor (**SR**) results in longer fluorescence lifetime of the donor (**SF**); see the unbleached (a) and photobleached (b) areas. These changes in fluorescence lifetimes are accompanied by changes in the fluorescence intensities (Figures 7B′ and 7C′).

## Conclusion

We demonstrated a new route for the formation of optically functional crystals. Size‐selective inclusion of chromophores in differently sized nanosized channels in MOFs has been demonstrated. The resulting crystals contain two different chromophores in closely spaced, parallel channels. Each type of channel contains only one of the two chromophores. The scope of this new process has been demonstrated by using pairs of different chromophores. The spatially resolved host–guest micro‐sized structures display anisotropic optical properties. The physically separated arrays of chromophores interact as demonstrated by FRET, which is a function of the concentration of the acceptor included in the crystal. Photo‐bleaching experiments on single crystals confirmed the energy transfer between the arrays of different dyes and provide opportunities to generate patterned microscale structures that have areas with different fluorescence characteristics. Our findings can be extended to other dyes and MOFs that have differently‐sized channels. Combining the anisotropic optical properties with FRET can find applications in optical switches and as bulk materials for light harvesting and up‐conversion.

## Conflict of interest

The authors declare no conflict of interest.

1

## Supporting information

As a service to our authors and readers, this journal provides supporting information supplied by the authors. Such materials are peer reviewed and may be re‐organized for online delivery, but are not copy‐edited or typeset. Technical support issues arising from supporting information (other than missing files) should be addressed to the authors.

Supporting Information

Supporting Information

Supporting Information

Supporting Information

Supporting Information

Supporting Information

Supporting Information

## Data Availability

The data that support the findings of this study are available in the supplementary material of this article.
