# Supplementary material for: Energy Transport in Dichroic Metallo‐organic Crystals: Selective Inclusion of Spatially Resolved Arrays of Donor and Acceptor Dyes in Different Nanochannels
Source: Angew Chem Int Ed Engl. 2022 Dec 16;62(4):e202214041. doi: 10.1002/anie.202214041 (PMC10107947; doi:10.1002/anie.202214041)
Supplement: Supplementary file 1 — Supporting Information [file ANIE-62-0-s007.pdf]

Angewandte Chemie
Eine Zeitschrift der Gesellschaft Deutscher Chemiker

Supporting Information

Energy Transport in Dichroic Metallo-organic Crystals: Selective Inclusion of Spatially Resolved Arrays of Donor and Acceptor Dyes in Different Nanochannels

Q. Wen, N. Malik, Y. Addadi, M. Weißenfels, V. Singh, L. J. W. Shimon, M. Lahav, M. E. van der Boom**

Supporting Information

Energy Transport in Dichroic Metallo-organic Crystals: Selective Inclusion of Spatially Resolved Arrays of Donor and Acceptor Dyes in Different Nanochannels

Qiang Wen[†], Naveen Malik[†], Yoseph Addadi[#], Maren Weißenfels[†], Vivek Singh[†], Linda J. W. Shimon[‡], Michal Lahav[†,*], and Milko E. van der Boom[†,*]

[†]*Department of Molecular Chemistry and Materials Science,* [#]*Department of Life Science Core Facilities,* [‡]*Department of Chemical Research Support, Weizmann Institute of Science, Rehovot 7610001, Israel. Email: michal.lahav@weizmann.ac.il; milko.vanderboom@weizmann.ac.il*

TABLE OF CONTENTS

EXPERIMENTAL SECTION

MATERIALS AND METHODS

S5	Optical microscopy
S5	UV/Vis spectroscopy
S5	Scanning electron microscopy (SEM)
S5	Powder X-ray diffraction (PXRD) measurements
S6	Fluorescence lifetime imaging microscopy (FLIM)
S6	Steady-state fluorescence spectroscopy

EXPERIMENTAL PROCEDURES

S7	Preparation of crystals
S7	Quantification of the amounts of chromophores embedded in the crystals
S8	Stepwise inclusion of two differently-sized dyes: sodium fluorescein (**SF**) and sodium resorufin (**SR**)
S8	Crystals saturated with sodium fluorescein (**SF**) and different amounts of sodium resorufin (**SR**)
S8	Sodium resorufin (**SR**), methyl orange (**MO**), or 7,7,8,8-tetracyanoquinodimethane lithium salt (**TCNQ**) inclusion in crystals saturated with sodium fluorescein (**SF**)
S9	Single-crystal X-ray diffraction analysis

FIGURES AND TABLES

S10	**Figure S1.** Optical microscope images showing hexagonal plates observed as a minor product (<2%).
S10	**Figure S2.** X-ray crystal structure of a hexagonal plate and side view of two different channels (CIF v370; CCDC 2131621). The channel diameters are 1 nm and 0.7 nm.
S11	**Table S1.** Crystal data and structural refinement.

S12-S14 **Figure S3.** Demonstration of dye inclusion from methanol solutions into the crystals.

S15-S17 **Figure S4.** Quantification of the amounts of chromophores embedded in the crystals.

S18 **Figure S5.** Calibration curve for quantifying the amount crystals used for dye inclusion.

S18 **Table S2.** Quantification of dye uptake by the crystals from methanol solutions.

S19 **Figure S6.** Sequence-dependent and size-selective functionalization of the different nanosized channels of the host crystals with the sodium salts of fluorescein (**SF**) and resorufin (**SR**).

S20 **Figure S7.** Photographs of the vials with crystals saturated with both sodium fluorescein (**SF**) and sodium resorufin (**SR**) in methanol.

S21 **Figure S8.** Sodium resorufin (**SR**), methyl orange (**MO**), or 7,7,8,8-tetracyanoquinodimethane lithium salt (**TCNQ**) inclusion in crystals saturated with sodium fluorescein (**SF**).

S22 **Figure S9.** Optical images of crystals loaded with sodium fluorescein (**SF**, 0.03 mmol/g) and the lithium salt of 7,7,8,8-tetracyanoquinodimethane (**TCNQ**, 0.05 mmol/g).

S23 **Figure S10.** Size-selective functionalization of the different nanosized channels of the host crystals with the sodium salts of fluorescein isothiocyanate isomer I (**FITC**) and resorufin (**SR**).

S24 **Figure S11.** Fluorescence spectra of sodium fluorescein (**SF**) and sodium resorufin (**SR**).

S25 **Figure S12.** Fluorescence lifetimes of the bulk and curve fitting. Demonstration of Förster resonance energy transfer (FRET) between the sodium salts of fluorescein (**SF**) and resorufin (**SR**) in the host crystals with confocal fluorescence lifetime imaging microscopy (FLIM).

S26 **Table S3.** Lifetime analysis and curve fitting of experimental data shown in **Figure 6** and **Figure S12**.

S26 **Table S4.** Energy transfer efficiency.

S27 **Figure S13.** Fluorescence lifetime images of crystals saturated with sodium fluorescein (**SF**) and with different amounts of sodium resorufin (**SR**).

S28 **Figure S14.** Fluorescence intensity images of crystals saturated with sodium fluorescein (**SF**) and with different amounts of sodium resorufin (**SR**).

S29 **Figure S15.** Z-scan images showing the fluorescence (A) intensity and (B) lifetime of crystals saturated with sodium fluorescein (**SF**). Scale bar: 20 μm.

S29 **Figure S16.** Z-scan images showing the fluorescence (A) intensity and (B) lifetime of crystals saturated with sodium fluorescein (**SF**, 0.03 mmol/g) and loaded with sodium resorufin (**SR1**, 3.7×10^{-5} mmol/g).

S30 **Figure S17.** Z-scan images showing the fluorescence (A) intensity and (B) lifetime of crystals saturated with sodium fluorescein (**SF**, 0.03 mmol/g) and loaded with sodium resorufin (**SR2**, 2.5×10^{-3} mmol/g).

S31 **Figure S18.** Z-scan images showing the fluorescence (A) intensity and (B) lifetime of crystals saturated with sodium fluorescein (**SF**, 0.03 mmol/g) and loaded with sodium resorufin (**SR3**, 4.9×10^{-3} mmol/g).

S32 **Figure S19.** Z-scan images showing the fluorescence (A) intensity and (B) lifetime of crystals saturated with sodium fluorescein (**SF**, 0.03 mmol/g) and loaded with sodium resorufin (**SR4**, 4.1×10^{-2} mmol/g).

S33 **Figure S20.** Fluorescence lifetimes of the bulk and curve fitting of experimental data shown in **Figure 7**.

S34 **Table S5.** Lifetime analysis and curve fitting of experimental data shown in **Figure 7** and **Figure S13**.

S35. **References**

EXPERIMENTAL SECTION

MATERIALS AND METHODS

The ligand (1,3,5,7-tetrakis(4-((*E*)-2-(pyridin-4-yl)vinyl)phenyl) adamantane; **AdDB**) was prepared according to a literature procedure[S1]. Sodium resorufin (**SR**), methyl orange (**MO**), 7,7,8,8-tetracyanoquinodimethane (**TCNQ**), sodium fluorescein (**SF**), fluoresceinamine isomer I (**AF**), fluorescein isothiocyanate isomer I (**FITC**), brilliant blue R250 (**BBR**), and solvents were purchased from Sigma-Aldrich. Sulforhodamine B sodium salt (also known as acid red 52; **AR**) was purchased from Alfa Aesar. The commercially available materials were used without further purification. For all dyes the sodium salts were used, with the exception of 7,7,8,8-tetracyanoquinodimethane (**TCNQ**). The lithium salt of **TCNQ** was prepared according to a literature procedure[S2].

Optical microscopy. Optical microscopy images were taken using a Nikon E600 Pol microscope equipped with a Nikon DS Fi1 camera.

UV/Vis spectroscopy. The UV/Vis spectra were obtained using Varian Cary 100 spectrophotometers (in double beam transmission mode).

Scanning electron microscopy (SEM). SEM measurements were performed using HRSEM ULTRA-55 ZEISS and HRSEM SUPRA-55 VP ZEISS instruments at an EHT voltage of 1.5 kV. The SEM samples were prepared by placing a drop of the MOFs dispersed in ethanol on a silicon substrate, and the solvent was allowed to evaporate.

Powder X-ray diffraction (PXRD) measurements. The measurements were carried out in the reflection geometry using a TTRAX III (Rigaku, Japan) diffractometer equipped with a rotating Cu anode operating at 50 kV and 200 mA and with a scintillation detector aligned at the diffracted beam after a bent Graphite monochromater. The $2\theta/\theta$ scans were performed under specular conditions in the Bragg-Brentano mode with variable slits and were scanned from 2 to 45 degrees of 2θ, with a step size of 0.02 degrees and a scan speed of 0.5 degree per minute.

Fluorescence lifetime imaging microscopy (FLIM). Fluorescence lifetime and intensity images of crystals loaded with sodium resorufin (**SR**) and sodium fluorescein (**SF**) were obtained using a Leica TCS SP8 STED equipped with a tunable (470−670 nm) pulsed white light laser (WLL; repetition rate = 78 MHz) (Leica Microsystems). A sample of crystals loaded with dyes was deposited on a glass slide and covered with a cover slide. The magnetization of the objective is HC PL APO CS2 20×/0.75 DRY. Crystals loaded with **SF** were excited at λ = 470 nm and the fluorescence signals were collected between 500-540 nm for lifetime analysis. Crystals loaded with both **SR** and **SF** were excited at λ = 470 nm and the fluorescence signals were collected at λ = 500-540 nm (Channel 1) and λ = 650-700 nm (Channel 2). The pixel size was set to 0.191 μm. The final image size was 97.72 μm × 97.72 μm. The laser power was adjusted (0.02% - 0.07%) to avoid signal saturation. All analyses were done with Leica Microsystem software.

Steady-state fluorescence spectroscopy. Steady-state excitation and emission were conducted using a Varian Cary Eclipse fluorescence spectrophotometer operated in a synchronous scan mode. The excitation slit was set at 5 nm, and the emission slit was set to 2.5 nm. For the excitation-emission scan, the stepwise was set to 5 nm with a scanning speed of 120 nm/min. The photomultiplier gain was 800 V. The solid samples were prepared by putting a thin layer of crystals loaded with dyes between two glass slides. The slides were put an angle of ~ 45° between the light beam and the surface of a sample.

EXPERIMENTAL PROCEDURES

Preparation of crystals. The ligand (**AdDB**, 18.0 mg, 0.021 mmol) and $Cd(NO_3)_2 \times 4H_2O$ (50 mg, 0.16 mmol) were dissolved separately in DMF (6.0 mL and 5.0 mL, respectively). HCl (10 μL, 37%) was mixed with DMF (1.5 mL). Portions of these DMF solutions were added to a 20 mL fused-quartz vial: 1.0 mL of the solution containing **AdDB**, followed by the addition of the solutions containing HCl (0.5 mL), and $Cd(NO_3)_2 \times 4H_2O$ (0.5 mL). The closed vial was heated in an oven at 105 ℃ for 24 h. The oven was allowed to cool down to 25 ℃ by reducing the set temperature by 10 ℃ every hour. Crystals were collected by filtration under vacuum and washed with ethanol. A light-yellow powder was obtained after drying under vacuum (~10^{-2} mbar, 89% yield based on **AdDB**) (**Figure 1**, **S1, S2, Table S1**).

Quantification of the amounts of chromophores embedded in the crystals. Crystals (3-5 mg) were immersed in methanol (10 mL) for 24 h to remove residual DMF and acid, then washed with methanol (2×). The crystals were transferred into a cuvette and the dye solution in methanol was added. The dye uptake was followed for 24 h by UV/Vis spectroscopy measurements until saturation. The dye solutions (10 μL) were diluted with methanol to 1010 μL for quantification. The decrease in dye concentration in solution was used to quantify the amount of material absorbed by the crystals. The solution was removed and the crystals were washed with methanol (2×). The crystals were digested with a 2.0 mL solution of $CH_3OH/CHCl_3/CF_3COOH$, v/v/v = 10/25/1. Subsequently, the solution was analyzed by UV/Vis spectroscopy to quantify the exact amount of crystals based on the optical signature of the protonated ligand using a calibration curve (**Figure S4**, **S5**, **Table S2**).

Stepwise inclusion of two differently-sized dyes: sodium fluorescein (SF) and sodium resorufin (SR)

*Experiment 1: Loading of **SF** followed by **SR**.* The crystals were immersed in a 600 μL methanol solution containing **SF** (2.62×10^{-4} M). The dye uptake was followed by UV/Vis spectroscopy (uptake of **SF**: ~0.03 mmol/g). After saturation of the crystals with **SF**, 15 μL of a methanol solution containing **SR** (2.66×10^{-3} M) was added and the solution was further monitored by UV/Vis spectroscopy (uptake **SR** = 0.05 mmol/g) (**Figure 3A**,**B, S6**).

*Experiment 2: Loading of crystals with **SR** followed by attempted loading with **SF**.* The crystals were immersed in a 600 μL methanol solution containing **SR** (2.2×10^{-4} M). The dye uptake was followed by UV/Vis spectroscopy (uptake **SR** = 0.12 mmol/g). After saturation of the crystals with **SR**, 20 μL of a methanol solution containing **SF** (7.9×10^{-3} M) was added and the solution was further monitored by UV/Vis spectroscopy. The uptake of **SF** is nihil (**Figure 3C**,**D**).

Sodium resorufin (SR), methyl orange (MO), or 7,7,8,8-tetracyanoquinodimethane lithium (TCNQ) salt inclusion in crystals saturated with sodium fluorescein (SF)

The crystals were immersed in a 200 μL methanol solution containing **SF** (7.8×10^{-3} M). After 24 h, the solution was removed and the crystals were washed with methanol (2×). Subsequently, these crystals were immersed in methanol solutions containing **SR** (100 μL, 1.1×10^{-3} M), or methyl orange (**MO**) (50 μL, 3.6×10^{-3} M), or **TCNQ** (50 μL; 5.2×10^{-3} M). The dye uptake was determined by UV/Vis spectroscopy. The dye solutions (10 μL) were diluted with methanol to 1010 μL for quantification. Dye uptake: **SF** = 0.03 mmol/g; **SR** = 0.05 mmol/g; **MO** = 0.030 mmol/g; **TCNQ** = 0.05 mmol/g (**Figure S7**). For optical images of the crystals functionalized with both **SF** and **TCNQ** (**Figure S8)**.

Crystals saturated with sodium fluorescein (SF) and different amounts of sodium resorufin (SR)

To a 5.0 mL vial with crystals (~20 mg), 600 μL of a methanol solution containing **SF** (7.9×10^{-3} M) was added. After saturation (24 h), the solution was removed and the crystals were kept in methanol (5 mL) for 3.0 h. Then, the batch of the functionalized crystals were divided into four vials. A methanol solution containing **SR** (1.1×10^{-5} M) was added to three of these vials for 10 min, 60 min and 120 min. The fourth batch of crystals was immersed in a methanol solution containing **SR** (7.8×10^{-5} M) for 14 h. For all batches, the solution was removed and the crystals were washed with methanol (3×). The amount of loading for all crystals: **SF** = 0.03 mmol/g; **SR1**, t = 10 min: 3.7×10^{-5} mmol/g; **SR2**, t = 60 min: 2.5×10^{-3} mmol/g; **SR3**, t = 120 min: 4.9×10^{-3} mmol/g, **SR4**, t = 14 h: 4.1×10^{-2} mmol/g. These crystals were used for fluorescence measurements (**Figure 5-7, S10-S16**).

Single-crystal X-ray diffraction analysis

Crystals were coated in Paratone oil (Hampton Research) and mounted on MiTeGen loops. They were flash frozen in a liquid nitrogen stream of Oxford Cryostream. Diffraction data of the crystals were collected at a low temperature of 100(2) K using CuKα λ = 1.54184 Å on a Rigaku XtaLab^{Pro} diffractometer equipped with a Dectris Pilatus 3R 200K-A detector for v370 and v375. The Rigaku data were processed and reduced with CrysAlisPro 1.171.39.46 (Rigaku OD, 2018). The structure was solved with SHELXT-2016/4 and refined with SHELXL-2016/4[S3].All non-hydrogen atoms were refined anisotropically and hydrogens were placed in calculated positions and refined in riding mode. The SQUEEZE protocol of Platon was run on all structures[S4]. Diffraction data of the colorless prism (v530) were collected at a low temperature of 100(2) K using CuKα λ = 1.54184 Å on a Rigaku XtaLAB Synergy R rotating anode system equipped with an HyPix-Arc 150 detector. The Rigaku data were processed and reduced with 'CrysAlisPro 1.171.41.111a (Rigaku OD, 2021)'. The structure could not be solved by direct methods so a starting model based on the solved colorless plates (v370) was used. These coordinates were refined against the data of v530 and fully refined in SHELXL-2016/4[S3]. All non-hydrogen atoms were refined anisotropically and hydrogens were placed in calculated positions and refined in riding mode. The SQUEEZE protocol of Olex2 was run[S5]. The crystal data and the structural refinement are summarized in **Figure 1**, **S2** and **Table S1**.

FIGURES AND TABLES

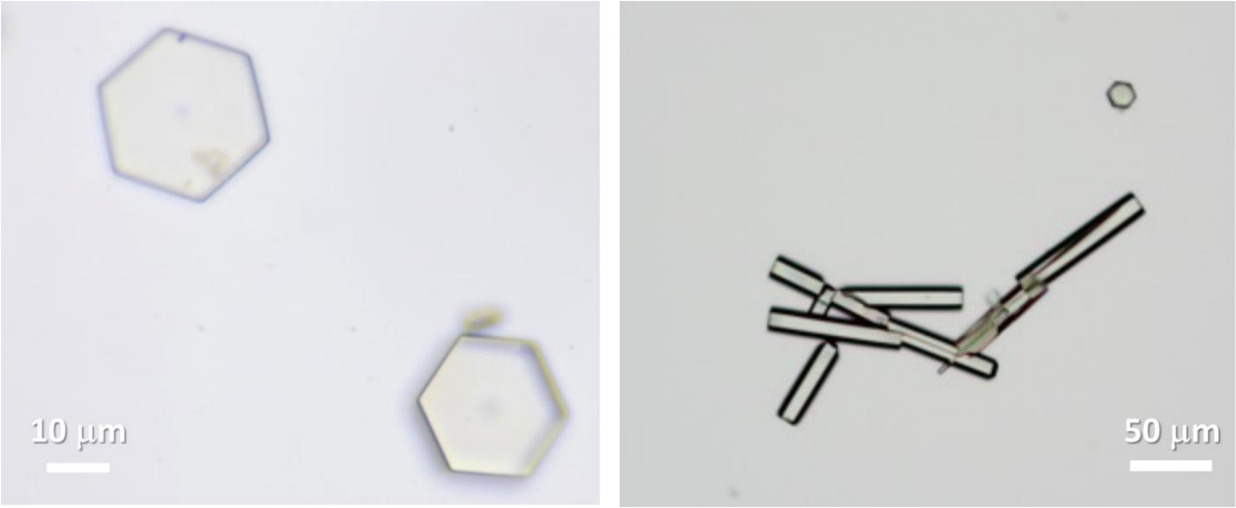

Figure S1. Optical microscope images showing hexagonal plates observed as a minor product (<2%).

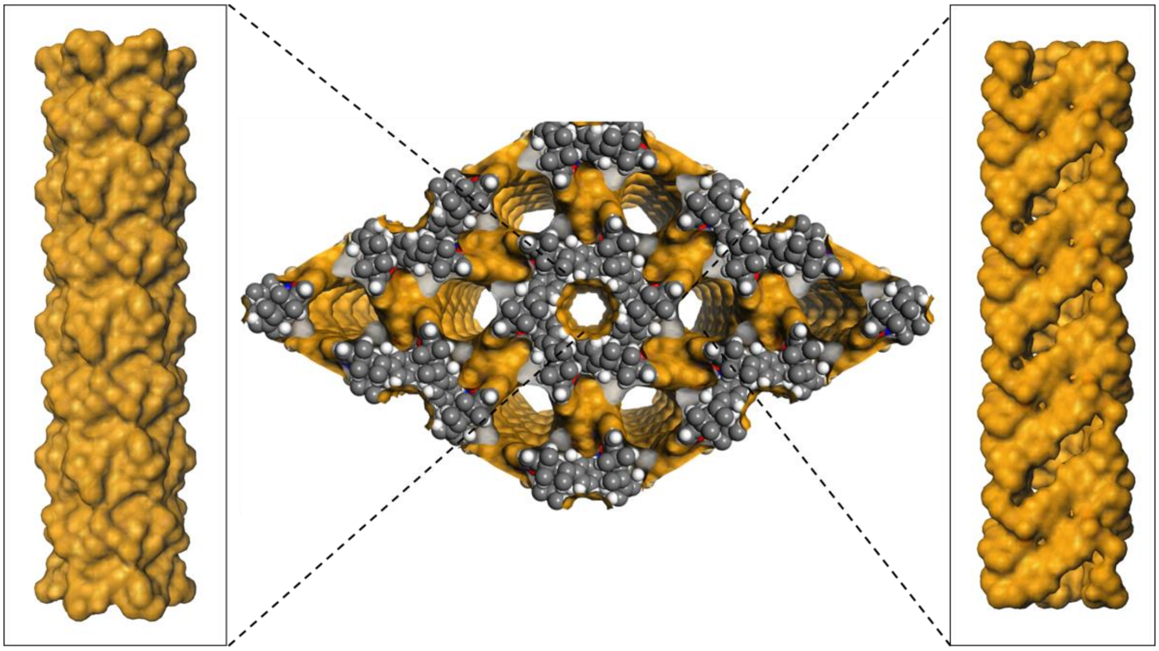

Figure S2. X-ray crystal structure of a hexagonal plate and side view of two different channels (CIF v370; CCDC 2131621). The channel diameters are 1 nm and 0.7 nm.

Table S1. Crystal data and structural refinement

Measurement name	v370	v375os[a]	v530
CCDC	2131621	2131622	2181332
Crystal description	Colorless plate	Yellow prism	Colorless prism
Crystal size (mm)	0.140 × 0.060 × 0.140	0.180 × 0.120 × 0.180	0.02 × 0.020 × 0.06
Diffractometer	Rigaku XtaLab^{Pro}	Rigaku XtaLab^{Pro}	Rigaku Synergy R
Empirical formula	$C_{62}H_{52}CdN_4O_2$ +[solvent]	$C_{62}H_{54}CdClN_4O_2$ + [solvent]	$C_{62}H_{52}CdClN_4Cl_2$ + [solvent]
Formula weight (g/mol)	1001.5	1018.94	1036.37
Temperature (K)	100	100	100
Wavelength (Å)	1.54184	1.54184	1.54184
Crystal system	hexagonal	hexagonal	hexagonal
Space group	*P622*	*P622*	*P622*
a (Å)	26.1666(8)	26.3679(5)	26.4235(4)
b (Å)	26.1666(8)	26.3679(5)	26.4235(4)
c (Å)	18.2494(5)	18.1682(3)	18.0234(3)
α,β, γ°	90,90,120	90,90,120	90,90,120
Volume (Å^3)	10821.2(7)	10939.4(5)	10898.0(4)
Z	6	6	6
Density calculated (Mg/m^3)	0.922	0.928	0.947
Absorption coefficient (mm^{-1})	2.684	2.981	3.319
F(000)	3120	3162	3204
Theta range for data collection (°)	2.421 to 61.863	4.565 to 77.246	3.345 to 55.99
Reflection collected (Unique)	22758 (5607)	29471 (7486)	204629 (4675)
R_{int}	0.0285	0.0480	0.0440
Completeness %	99.3	97.7	97.6
Index range *h*, *k*, *l*	-12, 23;-29,13, -20,19	-27, 33;-25,27;-21,21	-28, 28;-28,28;-17,17
Data/restraints/parameters	5607 / 151 / 279	7486 / 58 / 299	4675 / 7 / 265
Goodness-of-fit on F^2	1.035	1.060	1.191
Final R [I>2σ(I)]	R1=0.0853 wR2=0.2482	R1=0.0662 wR2=0.1969	R1=0.0904 wR2=0.2671
R (all data)	R1=0.1049 wR2=0.2749	R1=0.0785 wR2=0.2090	R1=0.1049 wR2=0.2897
Absolute structure parameter	0.28(3)	0.001(8)	0.075(14)
Helicity	*M*	*P*	*M*

[a] This crystal was saturated with the sodium salt of fluorescein (**SF**).

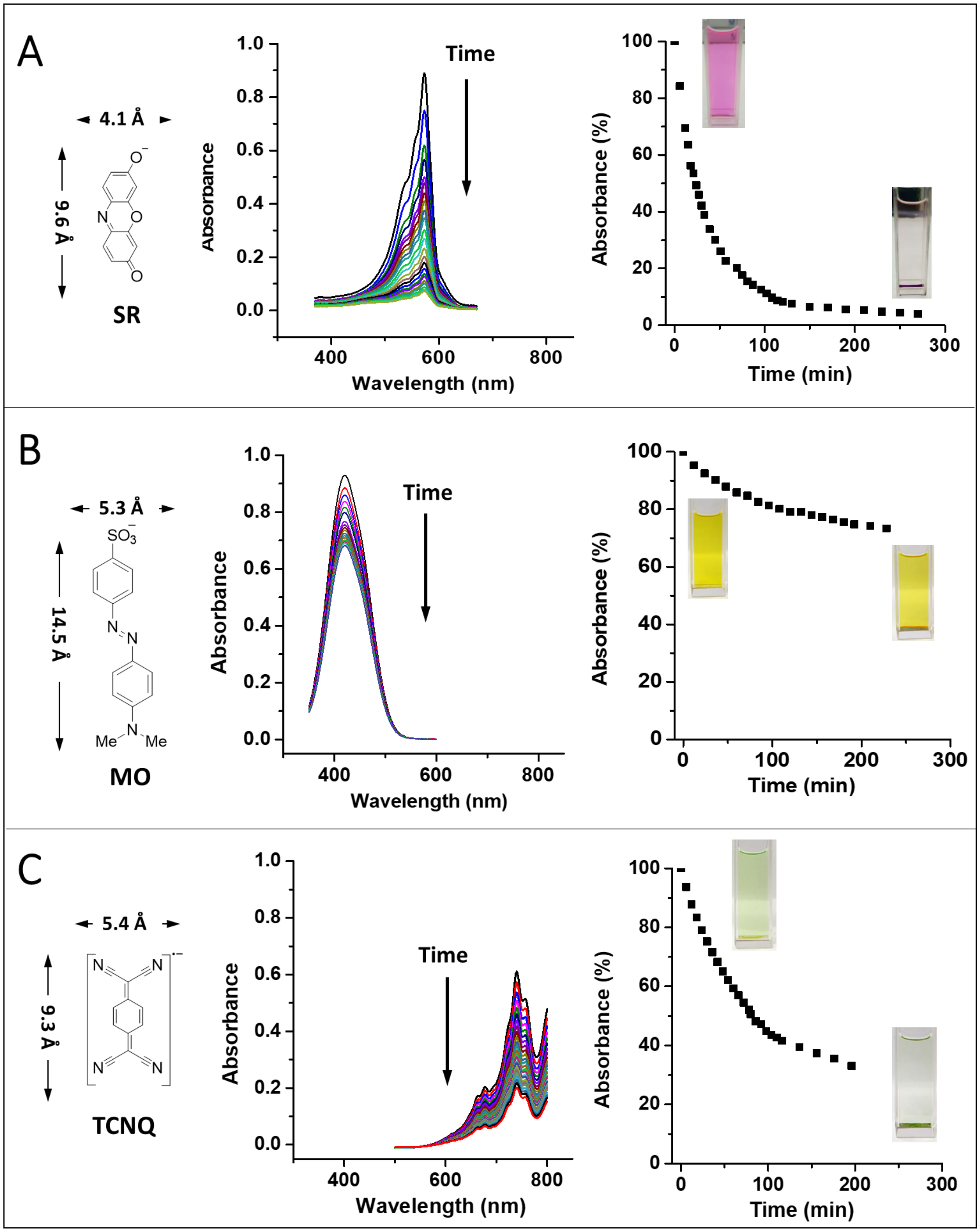

Figure S3. Demonstration of dye inclusion from methanol solutions into the crystals. Follow-up UV/Vis spectroscopy measurements. The sodium salts of (A) resorufin (**SR**) (600 μL MeOH, 8.9 × 10^{-5} M, λ_{max} = 572 nm), and (B) methyl orange (**MO**) (800 μL MeOH, 8.8 × 10^{-5}, λ_{max} = 420 nm), and the lithium salt (C) of 7,7,8,8-tetracyanoquinodimethane (**TCNQ**) (800 μL MeOH, 7.1 × 10^{-5} M, λ_{max} = 739 nm) were used.

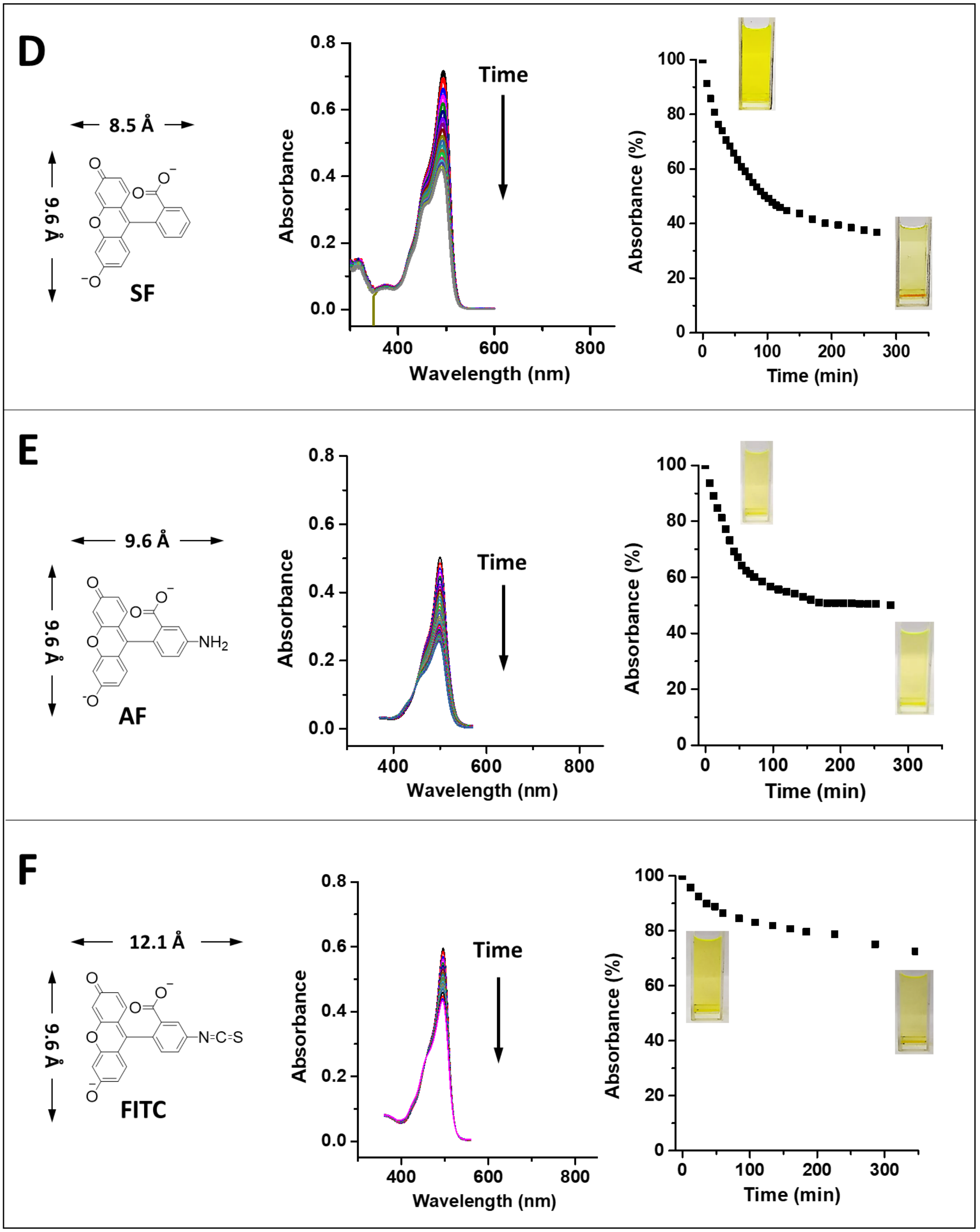

Figure S3 (Contd.) Demonstration of dye inclusion from methanol solutions into the crystals. Follow-up UV/Vis spectroscopy measurements. The sodium salts were used of (D) fluorescein (**SF**) (2.62×10^{-4} M, λ_{max} = 493 nm), (E) 5-aminofluorescein (**AF**) (0.86×10^{-5} M, λ_{max} = 499 nm), and (F) fluorescein isothiocyanate isomer I (**FITC**) (0.78×10^{-4} M, λ_{max} = 496 nm).

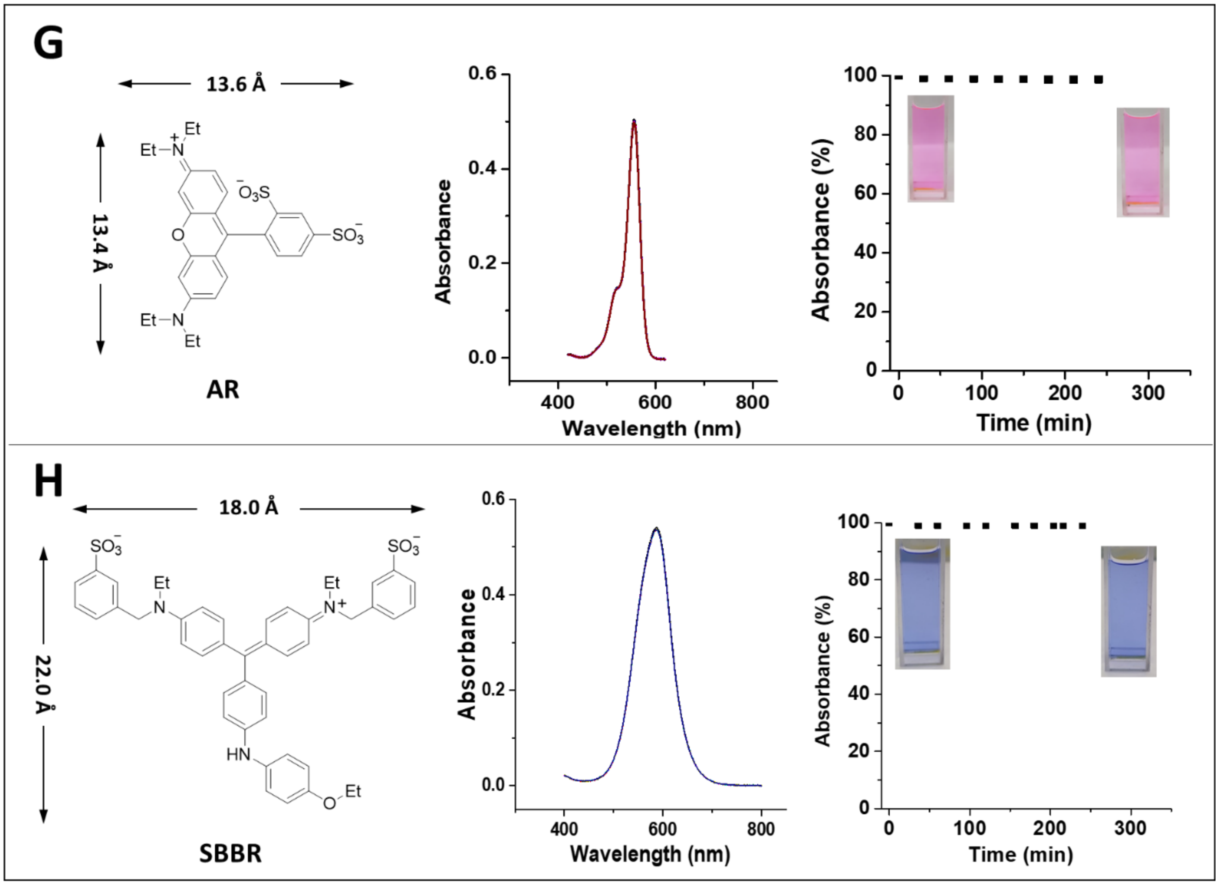

Figure S3 (Contd.) Demonstration of dye inclusion from methanol solutions into the crystals. Follow-up UV/Vis spectroscopy measurements. Note that the UV/Vis spectra overlap. The sodium salts of (G) sulforhodamine B (**AR**) (0.31×10^{-4} M, λ_{max} = 587 nm) and (H) brilliant blue R250 (**BBR**) (0.78×10^{-4} M, λ_{max} = 587 nm) were used.

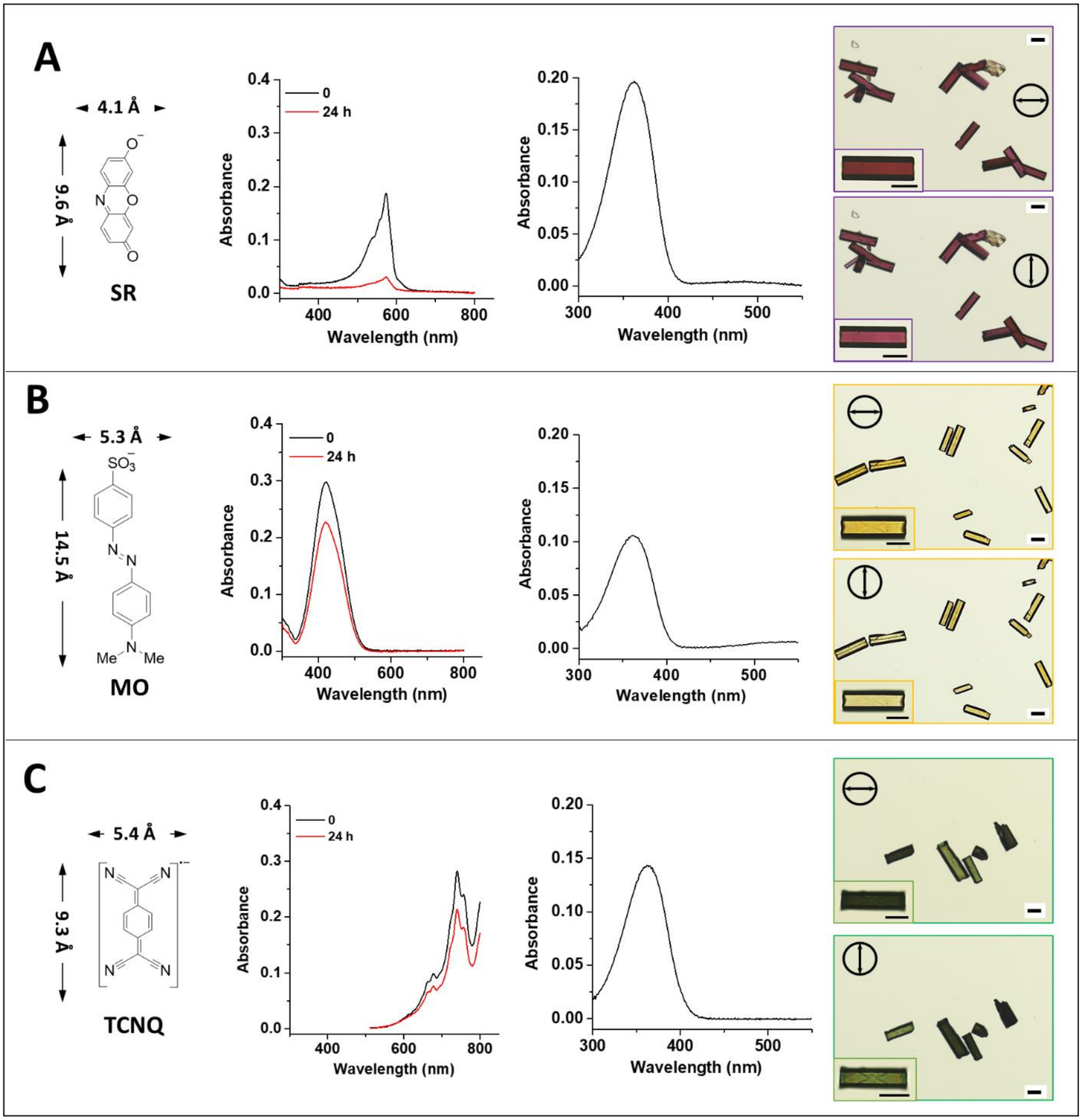

Figure S4. Quantification of the amounts of chromophores embedded in the crystals. Left: molecular structures of the sodium salts of: (A) resorufin (**SR**), (B) methyl orange (**MO**), and the lithium salt (C) 7,7,8,8-tetracyanoquinodimethane radial anion (**TCNQ**). Center: (i) the absorbance spectra of the chromophore-containing methanol solutions before (0 h, black) and after (24 h, red) adding the crystals, and (ii) the corresponding UV/Vis spectra of the digested dye-saturated crystals. Right: polarized light images of the colored crystals after 24 h reaction time. Scale bar = 50 μm. For details, see **Figure S5** and **Table S2**.

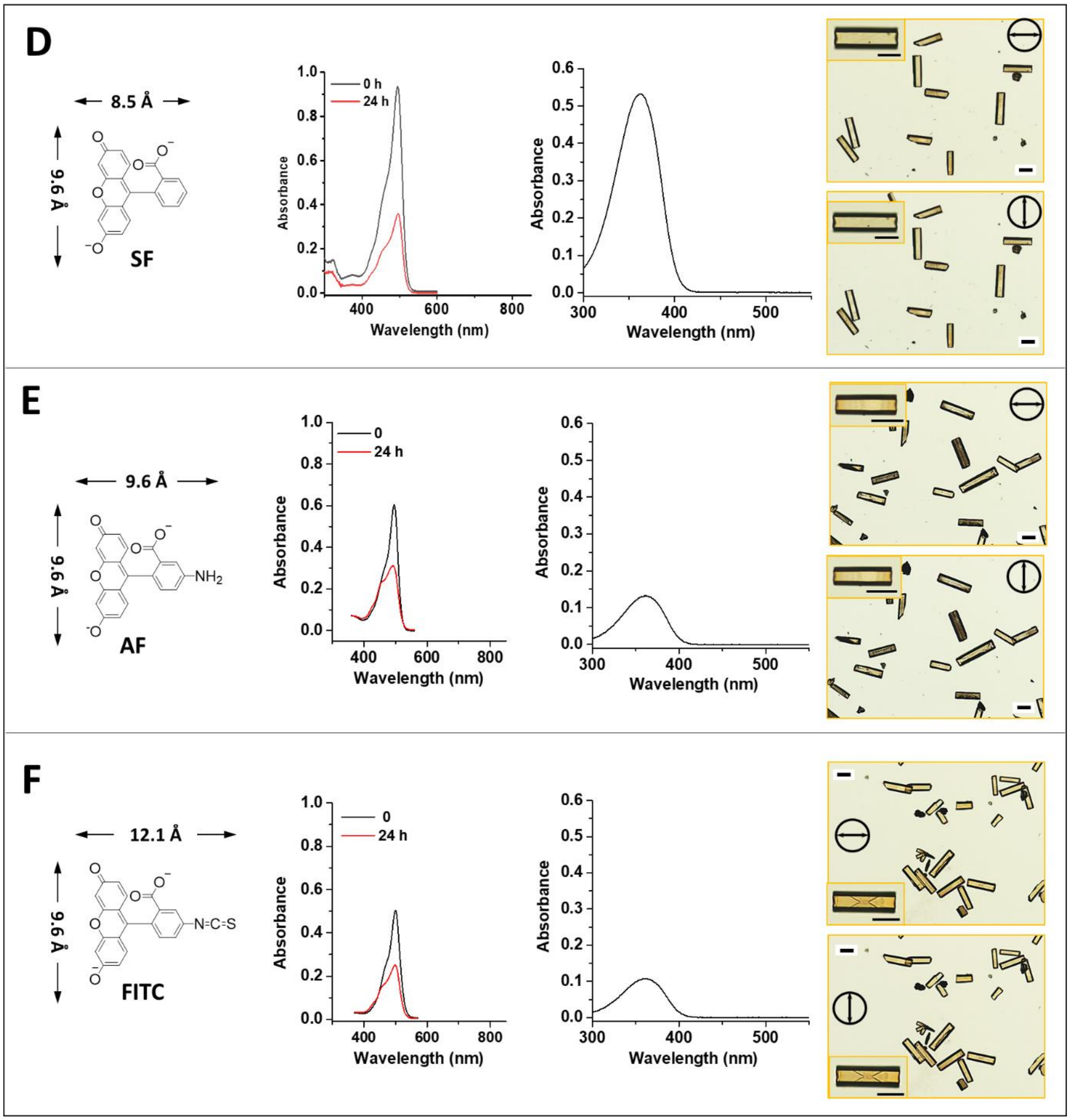

Figure S4 (Contd.). Quantification of the amounts of chromophores embedded in the crystals. Left: molecular structures of the sodium salts of: (D) fluorescein (**SF**), (E) 5-aminofluorescein (**AF**), (F) fluorescein isothiocyanate isomer I (**FITC**). Center: (i) the absorbance spectrum of the chromophore methanol solutions before (0 h) and after (24 h) adding the crystals, and (ii) the corresponding UV/Vis spectrum of the digested dye-saturated crystals. Right: polarized light images of the colored crystals after 24 h reaction time. Scale bar = 50 μm. For details, see **Figure S5** and **Table S2**.

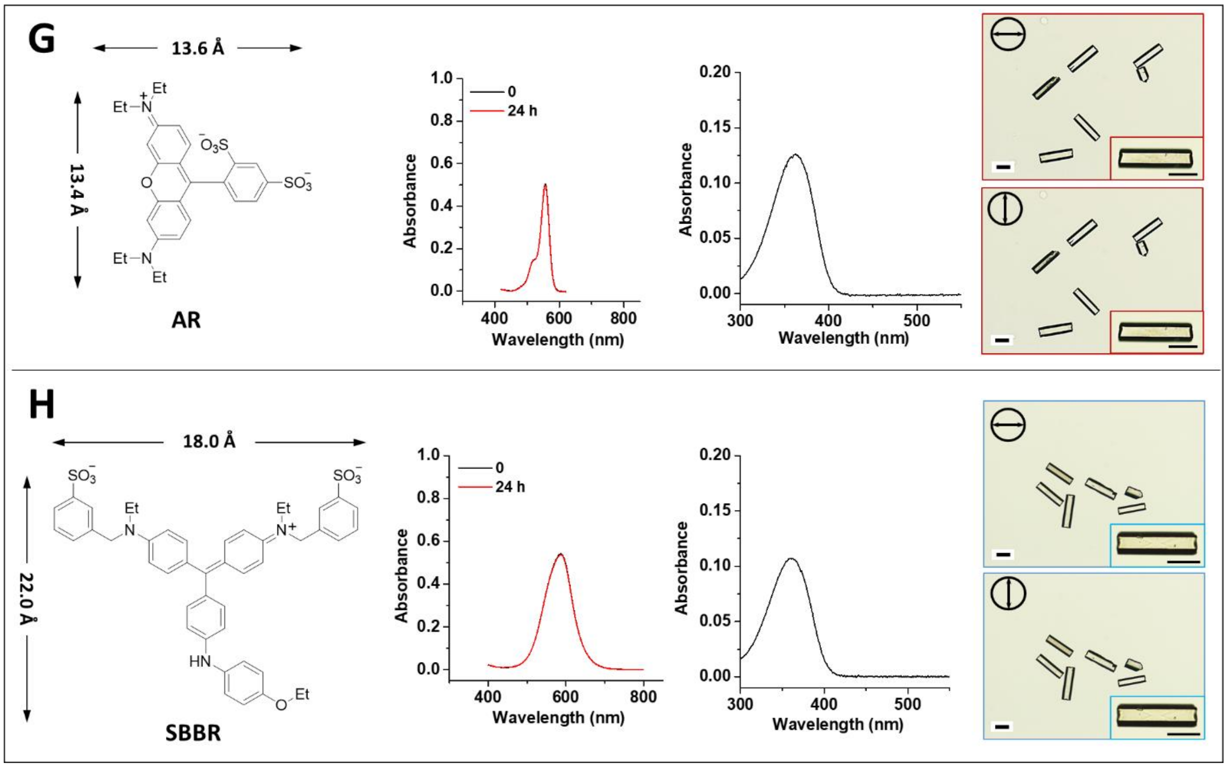

Figure S4 (Contd.). Quantification of the amounts of chromophores embedded in the crystals. Left: molecular structures of the sodium salts of: (A) Sulforhodamine B (**AR**) and (B) Brilliant blue R250 (**BBR**). Center: (i) the absorbance spectrum of the chromophore methanol solutions before (0 h) and after (24 h) adding the crystals, and (ii) the corresponding UV/Vis spectrum of the digested dye-saturated crystals, and polarized light images of the colored crystals after 24 h reaction time. Scale bar = 50 μm. For details, see **Figure S5** and **Table S2**.

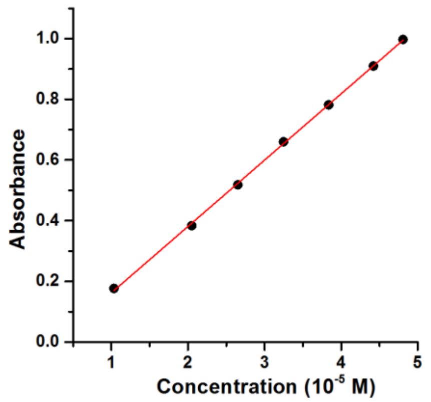

Figure S5. Calibration curve for quantifying the amount of crystals used for dye inclusion. This curve is obtained by UV/Vis measurements of seven solutions containing different concentrations of **AdDB** (3.0 mL of $CH_3OH/CHCl_3/CF_3COOH$, *v*/*v*/*v* = 10/25/1). The dots represent the intensity of the absorbance at λ_{max} = 361 nm *versus* the concentration of **AdDB**. The red line is the linear fit (y = a +b*x) with R^2 > 0.999.

Table S2 Quantification of dye uptake by the crystals from methanol solutions

Chromophore[a]		Uptake (mmol/g)[b]
**SR**	(1.1×10^{-3} M)	0.12
**MO**	(3.6×10^{-3} M)	0.11
**TCNQ**	(5.2×10^{-3} M)	0.14
**SF**	(7.9×10^{-3} M)	0.03
**AF**	(5.2×10^{-3} M)	0.02
**FITC**	(4.8×10^{-3} M)	0.02
**AR**	(1.9×10^{-3} M)	1.9×10^{-4}
**BBR**	(3.7×10^{-3} M)	5.3×10^{-4}

[a] The used concentrations of the chromophores in methanol. Sodium resorufin (**SR**), methyl orange (**MO**), 7,7,8,8-tetracyanoquinodimethane lithium salt (**TCNQ**), sodium fluorescein (**SF**), sodium fluoresceinamine isomer I (**AF**), sodium fluorescein isothiocyanate isomer I (**FITC**), sulforhodamine B sodium salt (**AR**), and brilliant blue R250 (**BBR**). [b] The uptake of the dyes normalized to the amount of crystals used. The di-anionic sodium forms of **SF**, **AF**, **FITC**, **AR** and **BBR** were prepared by adding 2.1 equivalent of sodium hydroxide (in MeOH) to the dye solutions. The solutions were stirred for 6 h and the formation of the di-anionic dyes was confirmed by UV/Vis spectroscopy.

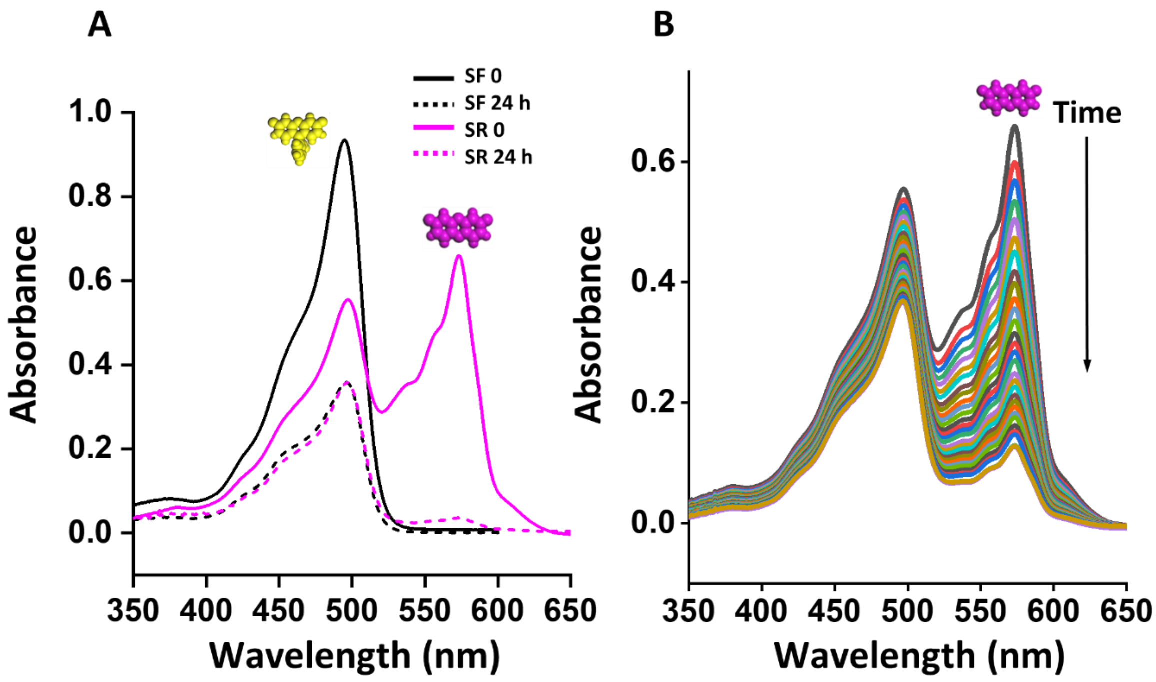

Figure S6. Sequence-dependent and size-selective functionalization of the different nanosized channels of the host crystals with the sodium salts of fluorescein (**SF**) and resorufin (**SR**). The process of the molecular sieving was monitored by UV/Vis spectroscopy. (A) Consecutive reactions of a suspension of the host crystals in methanol with **SF** and **SR** at t = 0 and t = 24 h. Dye uptake: **SF** = 0.03 mmol/g; **SR** = 0.05 mmol/g. (B) The same reaction of a methanol suspension of the host crystals saturated with **SF** with **SR**. The UV/Vis spectra show here the gradual uptake of the dyes (t = 0-24 h).

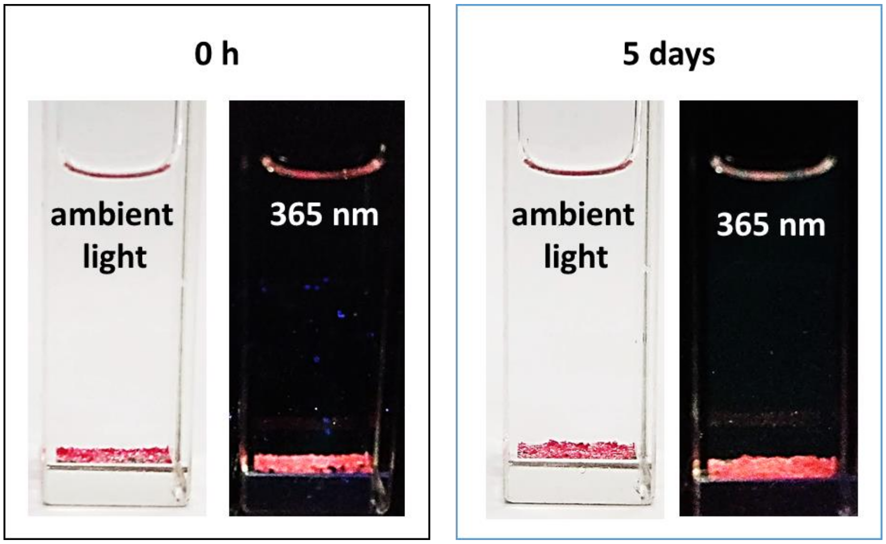

Figure S7. Photographs of the vials with crystals saturated with both sodium fluorescein (**SF**) and sodium resorufin (**SR**) in methanol, 0 h (left) and after 5 days (right).

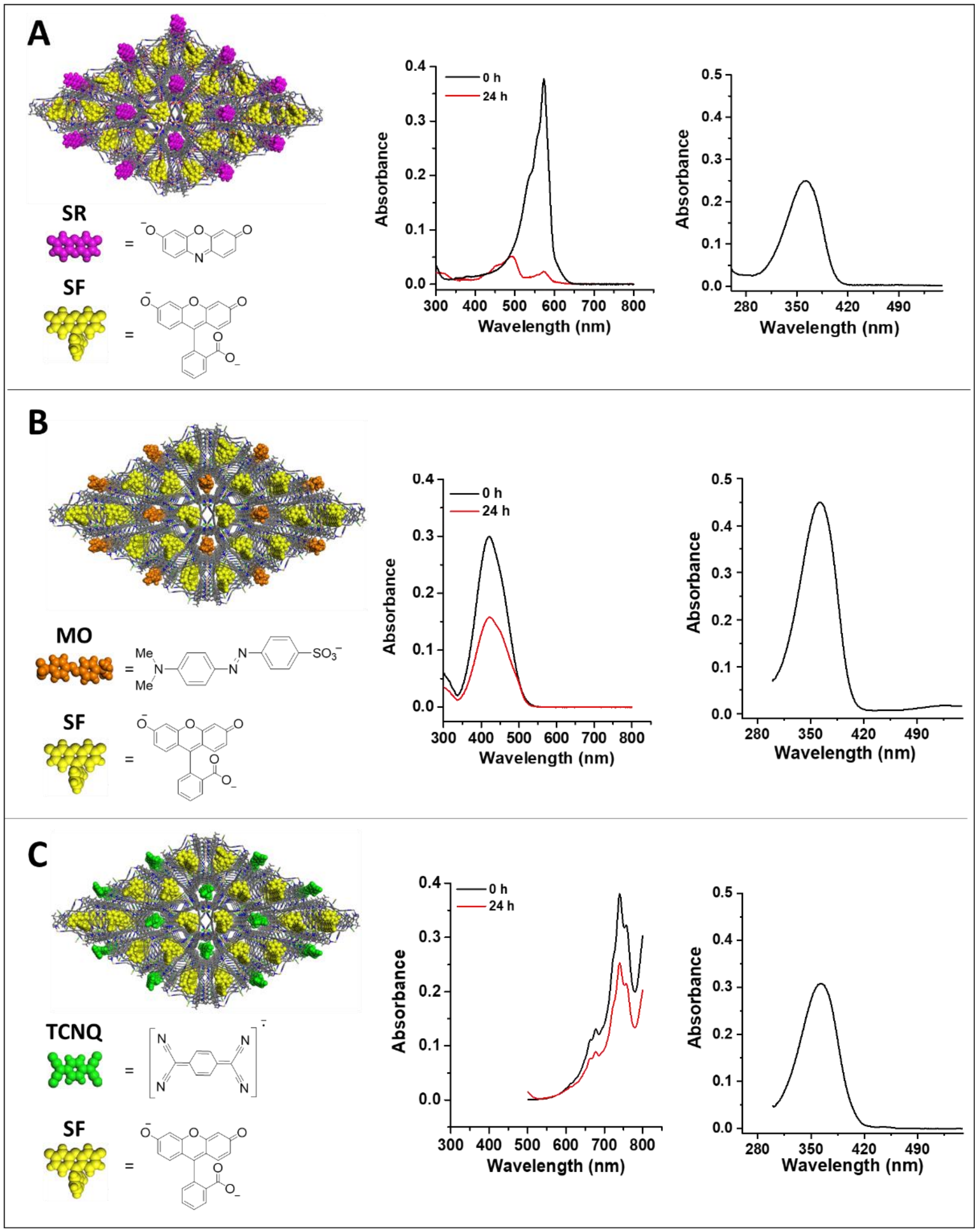

Figure S8. **Sodium resorufin (SR), methyl orange (MO), or 7,7,8,8-tetracyanoquinodimethane lithium salt (TCNQ) inclusion in crystals saturated with sodium fluorescein (SF).** Left: molecular structures of the dyes and proposed assembly. Center: the absorbance spectrum of the chromophore (**SR**, **MO** or **TCNQ**) methanol solutions before (0 h) and after (24 h) addition of the crystals. Right: the corresponding UV/Vis spectra of the solutions of the digested dye-saturated crystals. Dye uptake: (A) **SR** = 0.05 mmol/g; (B) **MO** = 0.03 mmol/g, (C) **TCNQ** = 0.05 mmol/g. For experimental details, see page S8.

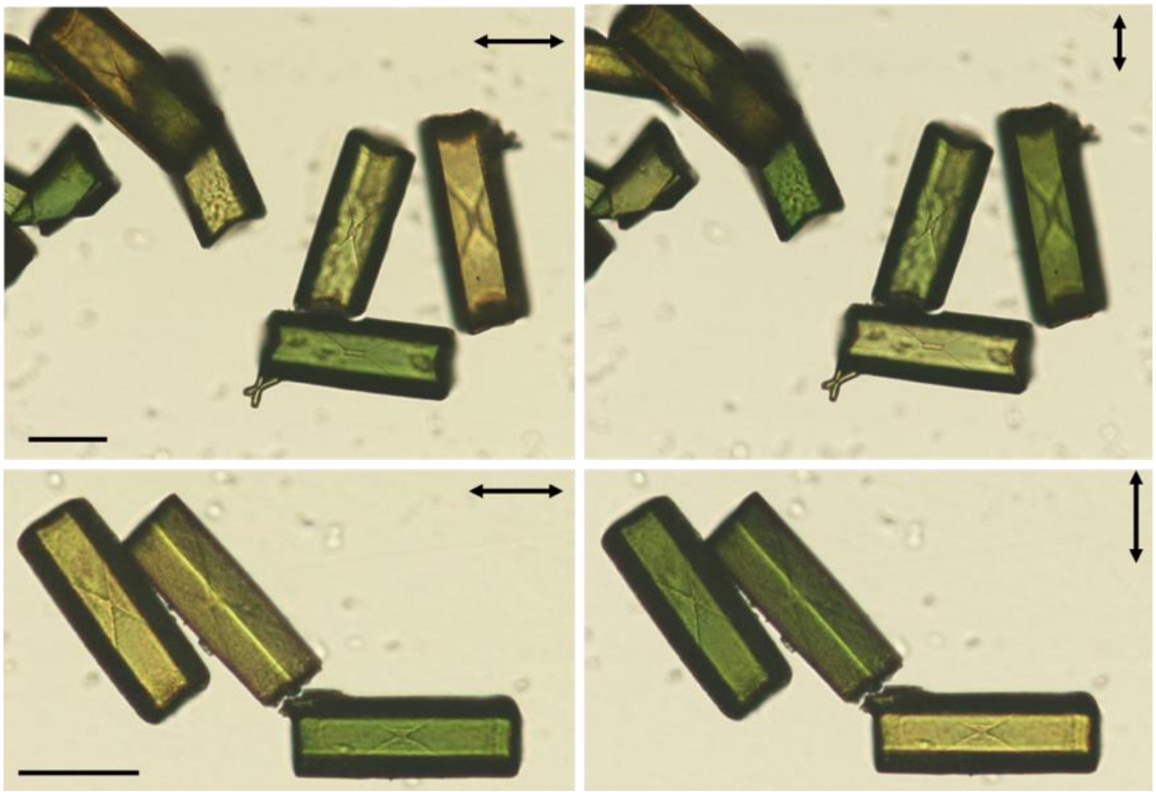

Figure S9. Optical images of crystals loaded with sodium fluorescein (**SF**, 0.03 mmol/g) and the lithium salt of 7,7,8,8-tetracyanoquinodimethane (**TCNQ**, 0.05 mmol/g). Scale bar: 50 μm. The polarization direction of light is indicated by arrow. For experimental details, see page S8.

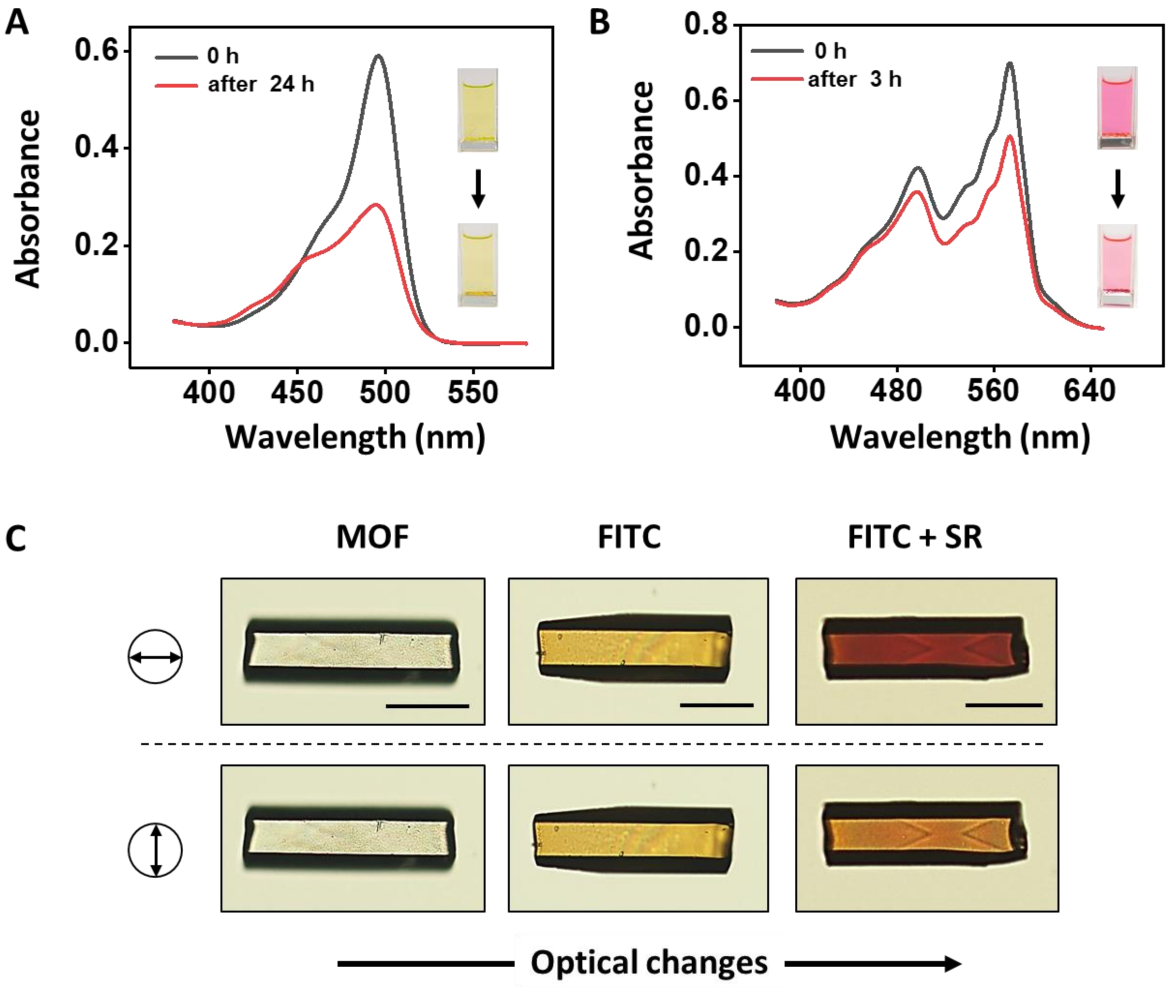

Figure S10. Size-selective functionalization of the different nanosized channels of the host crystals with the sodium salts of fluorescein isothiocyanate isomer I (**FITC**) and resorufin (**SR**). (A,B) Consecutive reactions of a suspension of the colorless host crystals in methanol (600 μL) with **FITC** (1.20×10^{-4} M; no change in absorbance was observed after 24 h) and **SR**. The latter dye was added in methanol (15 μL, 4.8×10^{-3} M) to the same quartz cell containing the solution with **FITC** and the host crystals saturated with **FITC**. Insets: The photos of the cuvettes show the color of the suspensions in the cuvettes at the beginning and at the end of the reactions. **FITC**: λ_{max} = 495 nm and **SR**: λ_{max} = 573 nm). Dye uptake: **FITC** = 0.02 mmol/g; **SR** = 0.008 mmol/g. (C) The optical images show the color of the crystals before and after the reactions with the dyes as a function of the incident angle (arrow) of polarized light. Scale bar = 50 μm.

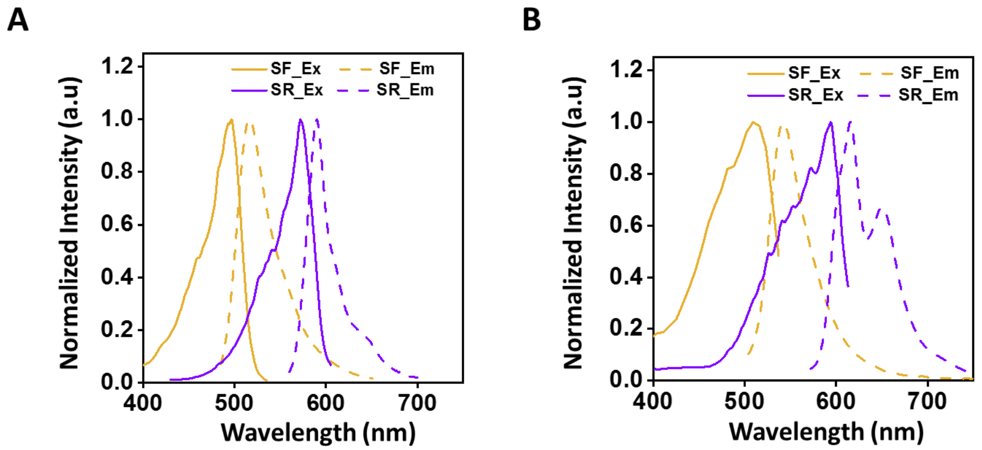

Figure S11. Fluorescence spectra of sodium fluorescein (**SF**) and sodium resorufin (**SR**). (A) Measurements of the dyes in solution. **SF**: 2.62 × 10^{-4} M (MeOH), λ_{ex} = 475 nm , λ_{em} = 545 nm; **SR**: 8.87 × 10^{-5} M (MeOH), λ_{ex} = 550 nm, λ_{em} = 615 nm. (B) Measurements of the dyes in the solid-state (bulk, included in the MOF). **SF**: 2.7 × 10^{-2} mmol/g, λ_{ex} = 470 nm, λ_{em} = 570 nm **SR**: 2.3 × 10^{-3} mmol/g. λ_{ex} = 560 nm, λ_{em} = 650 nm.

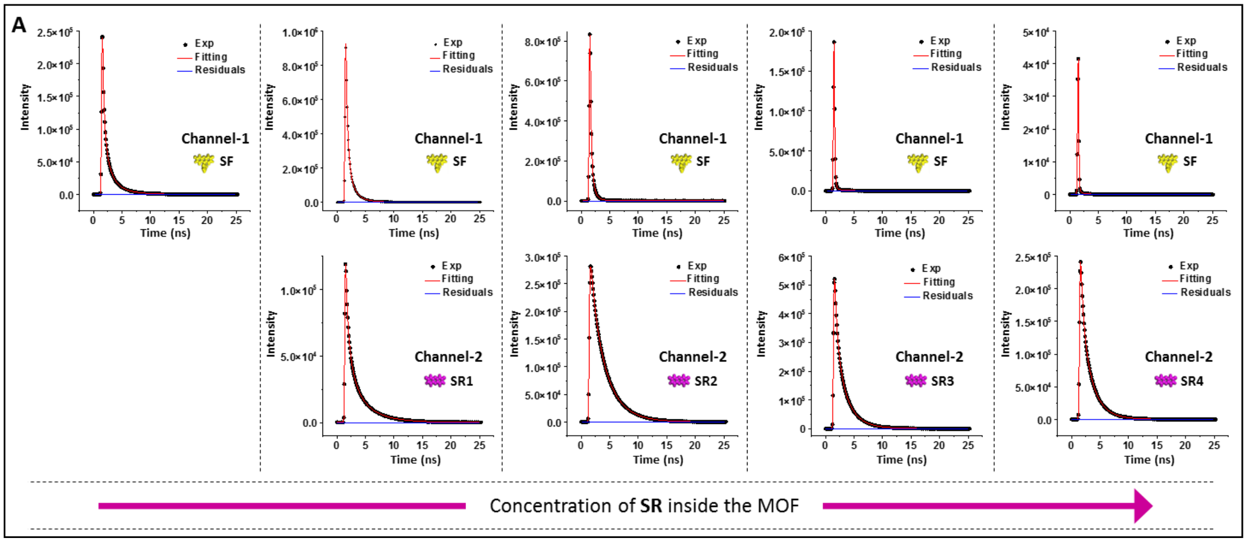

Figure S12. Fluorescence lifetimes of the bulk and curve fitting. Demonstration of Förster resonance energy transfer (FRET) between the sodium salts of fluorescein (**SF**) and resorufin (**SR**) in the host crystals with confocal fluorescence lifetime imaging microscopy (FLIM). The host crystals were saturated with **SF** (2.7 × 10^{-2} mmol/g) followed by functionalization with increasing amounts of **SR** (**SR1:** 3.7 × 10^{-5} mmol/g; **SR2**: 2.5 × 10^{-3} mmol/g; **SR3**: 4.9 × 10^{-3} mmol/g; **SR4**: 4.1 × 10^{-2} mmol/g). For further details, see **Figure 6** and **Table S3**.

Table S3. Lifetime analysis and curve fitting of experimental data shown in **Figure 6** and **Figure S12**. The fitted results are composed of three lifetime components (τ1, τ2, τ3). The average lifetime is also calculated including the intensity-weighted lifetime (τi) and amplitude-weighted lifetime (τa). Fluorescence lifetime imaging microscopy (FLIM) data analysis and fitting was performed using Leica application suite X (Leica Microsystems CNS GmbH) LAS X FLIM/FCS (Version 3.5.6).

	SF	SF+SR1		SF+SR2		SF+SR3		SF+SR4	
	Channel-1	Channel-1 (SF)	Channel-2 (SR1)	Channel-1 (SF)	Channel-2 (SR2)	Channel-1 (SF)	Channel-2 (SR3)	Channel-1 (SF)	Channel-2 (SR4)
Lifetime τ1 (ns)	0.259	0.295	0.330	0.188	0.403	0.094	0.378	0.068	0.374
Lifetime τ2 (ns)	1.069	0.966	1.223	0.582	1.803	0.516	1.391	0.651	1.543
Lifetime τ3 (ns)	3.005	2.608	3.623	2.121	3.113	2.958	2.628	2.460	2.595
Intensity weighted lifetime τi (ns)	1.477	0.941	2.569	0.423	2.692	0.394	1.863	0.360	1.806
Amplitude weighted lifetime τa (ns)	0.702	0.541	1.375	0.272	2.350	0.113	1.302	0.083	1.334
A1 (10^3)	205.005	871.613	64.690	1008.739	33.953	319.470	243.234	83.005	104.029
A2 (10^3)	100.101	324.870	48.053	235.320	123.348	7.381	257.412	0.966	142.852
A3 (10^3)	23.577	36.459	33.304	6.282	175.070	1.130	151.889	0.284	55.542
I1 (10^3)	546.954	2655.822	220.471	1954.810	141.191	309.946	948.325	58.184	401.279
I2 (10^3)	1103.070	3236.189	606.077	1412.779	2293.698	39.266	3692.960	6.481	2273.252
I3 (10^3)	730.339	980.672	1242.922	137.416	5618.448	34.456	4116.841	7.203	1486.079
χ^2	1.818	8.633	1.120	2.406	0.941	1.232	1.005	1.198	1.255

Table S4. Energy transfer efficiency.

	SF	SF+SR1	SF+SR2	SF+SR3	SF+SR4
	τD = 0.702	τDA = 0.541	τDA = 0.272	τDA = 0.113	τDA = 0.083
E = 1 – (τDA/τD)		23%	61%	84%	88%

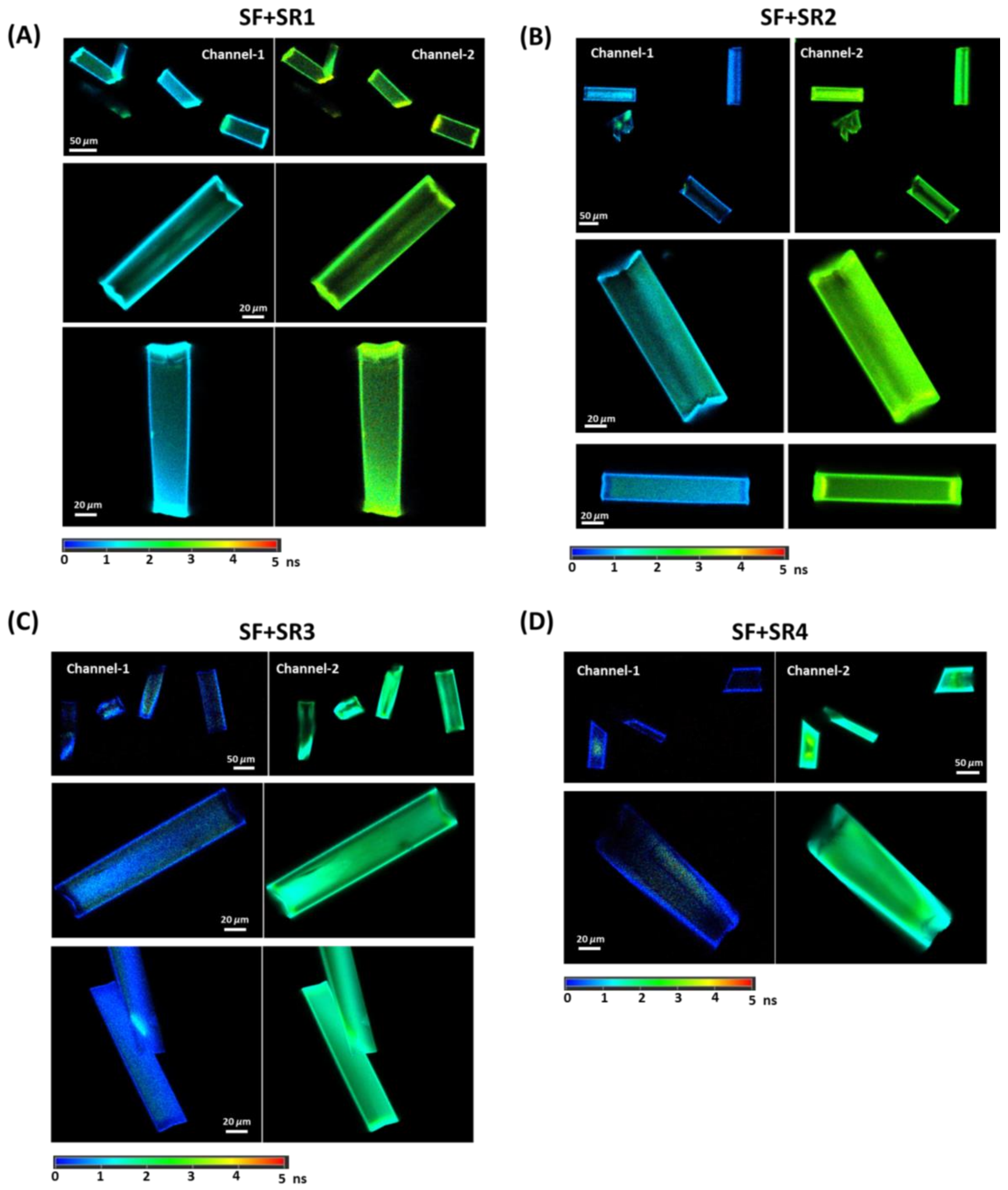

Figure S13. Fluorescence lifetime images of crystals saturated with sodium fluorescein (**SF**) and with different amounts of sodium resorufin (**SR**). The crystals were excited at λ = 470 nm and the signals were recorded at λ = 500-540 nm (left, Channel-1) and 650-700 nm (right, Channel-2). (A-D) **SF** = 0.03 mmol/g, (A) **SR1** = 3.7×10^{-5} mmol/g, (B) **SR2** = 2.5×10^{-3} mmol/g, (C) **SR3** = 4.9×10^{-3} mmol/g, (D) **SR4** = 4.1×10^{-2} mmol/g.

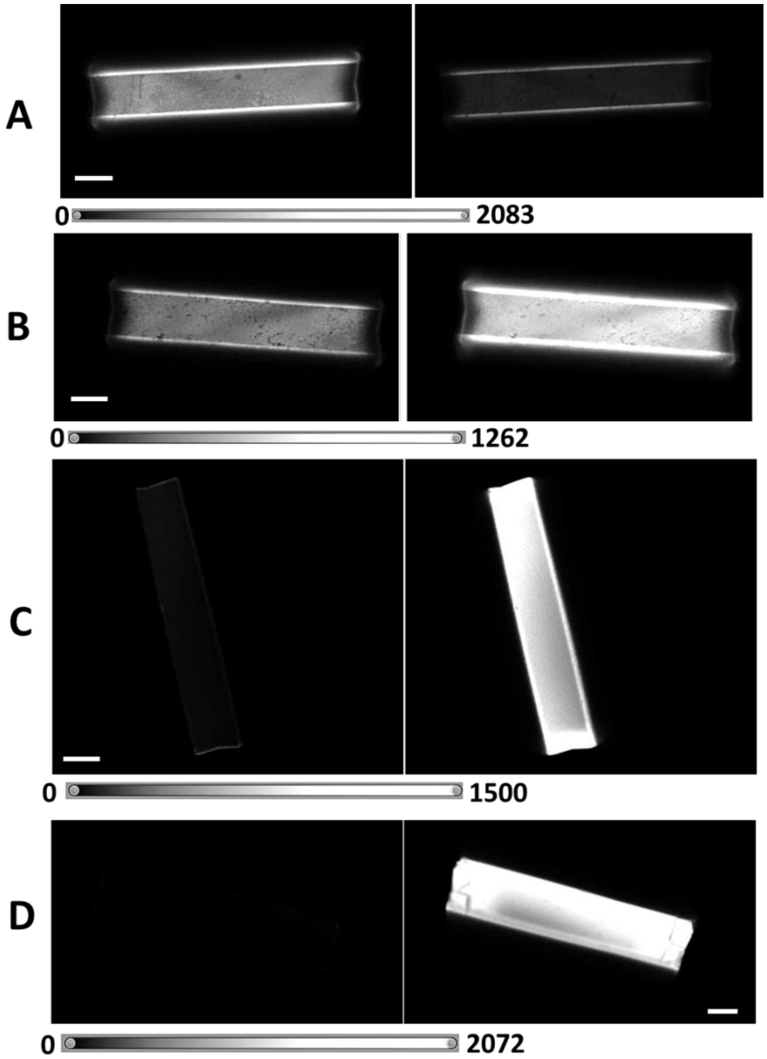

Figure S14. Fluorescence intensity images of crystals saturated with sodium fluorescein (**SF**) and with different amounts of sodium resorufin (**SR**). The crystals were excited at λ = 470 nm and the signals were recorded at λ = 500-540 nm (left) and λ = 650-700 nm (right). (A-D) **SF** = 0.03 mmol/g, (A) **SR1** = 3.7×10^{-5} mmol/g, (B) **SR2** = 2.5×10^{-3} mmol/g, (C) **SR3** = 4.9×10^{-3} mmol/g, (D) **SR4** = 4.1×10^{-2} mmol/g. Scale bar: 20 μm.

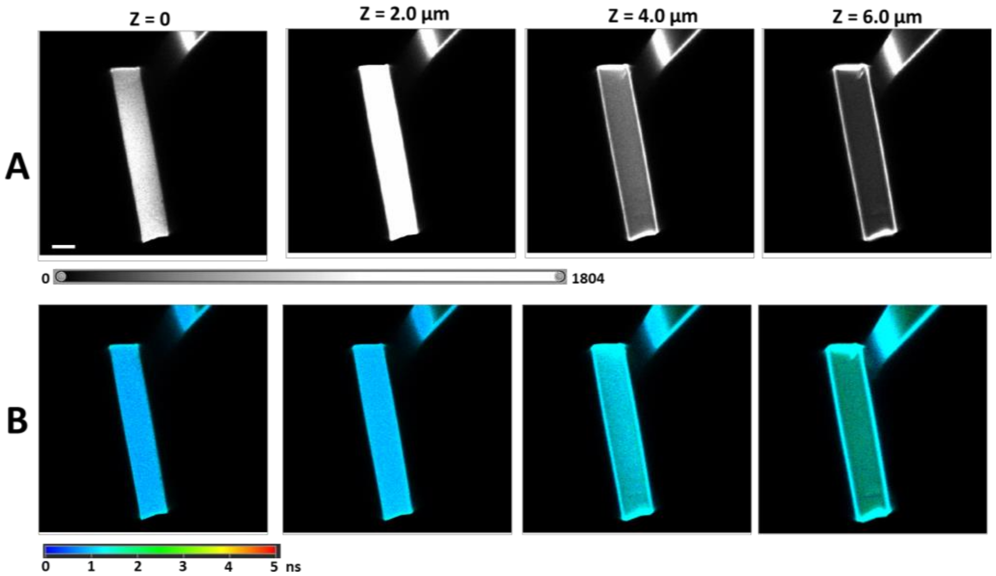

Figure S15. Z-scan images showing the fluorescence (A) intensity and (B) lifetime of crystals saturated with sodium fluorescein (**SF**, 0.03 mmol/g). Scale bar: 20 μm.

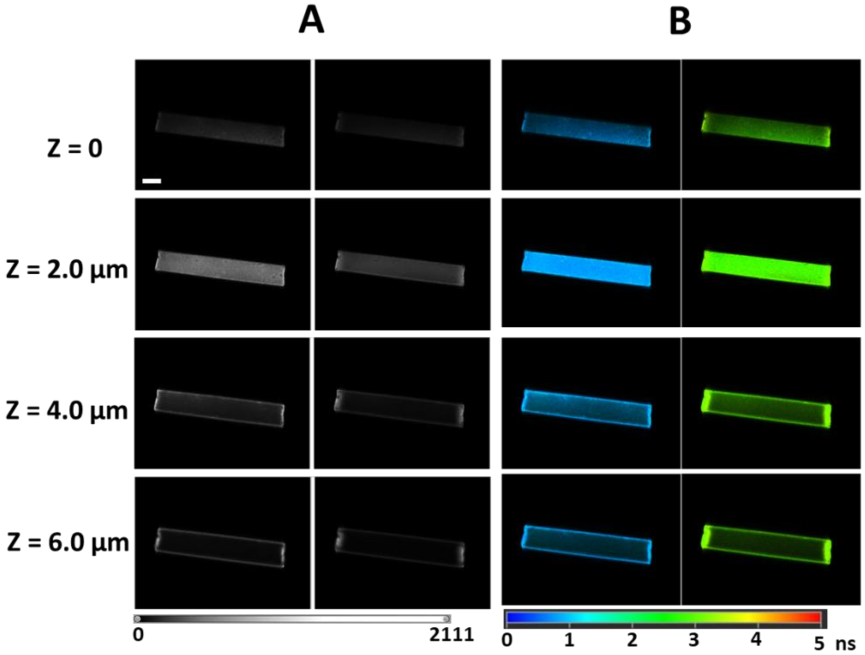

Figure S16. Z-scan images showing the fluorescence (A) intensity and (B) lifetime of crystals saturated with sodium fluorescein (**SF**, 0.03 mmol/g) and loaded with sodium resorufin (**SR1**, 3.7 × 10^{-5} mmol/g). Scale bar: 20 μm.

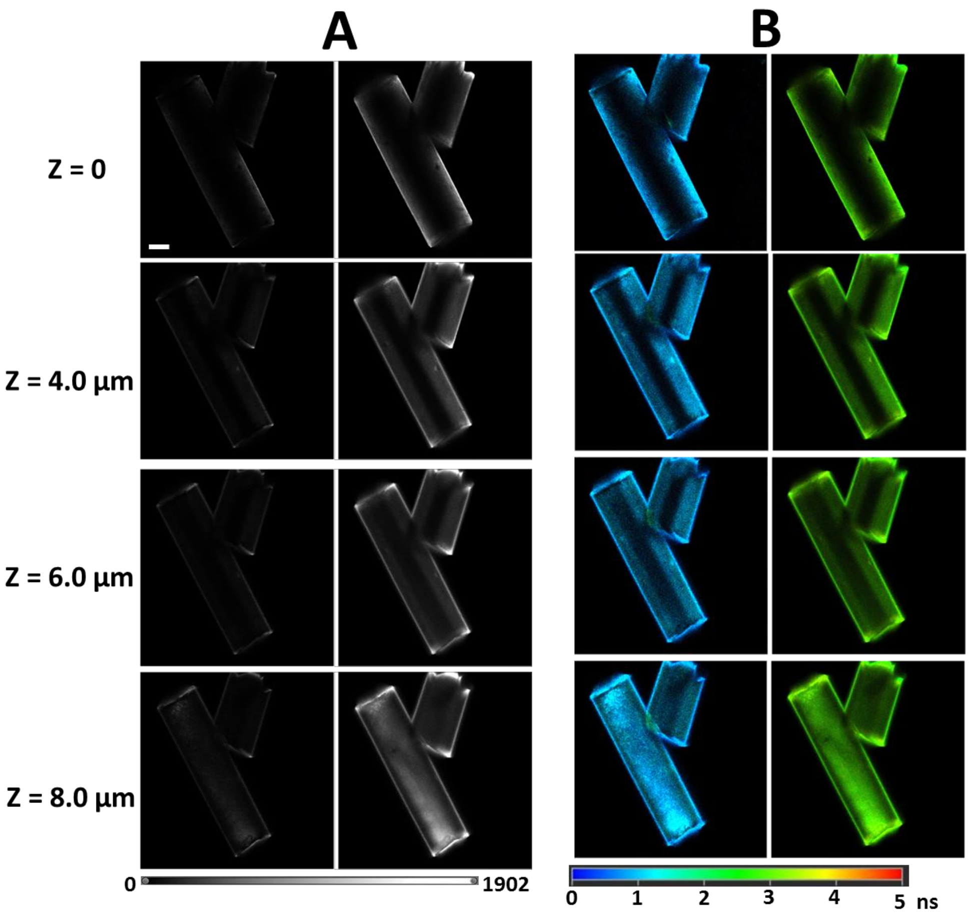

Figure S17. Z-scan images showing the fluorescence (A) intensity and (B) lifetime of crystals saturated with sodium fluorescein (**SF**, 0.03 mmol/g) and loaded with sodium resorufin (**SR2**, 2.5 × 10^{-3} mmol/g). Scale bar: 20 μm.

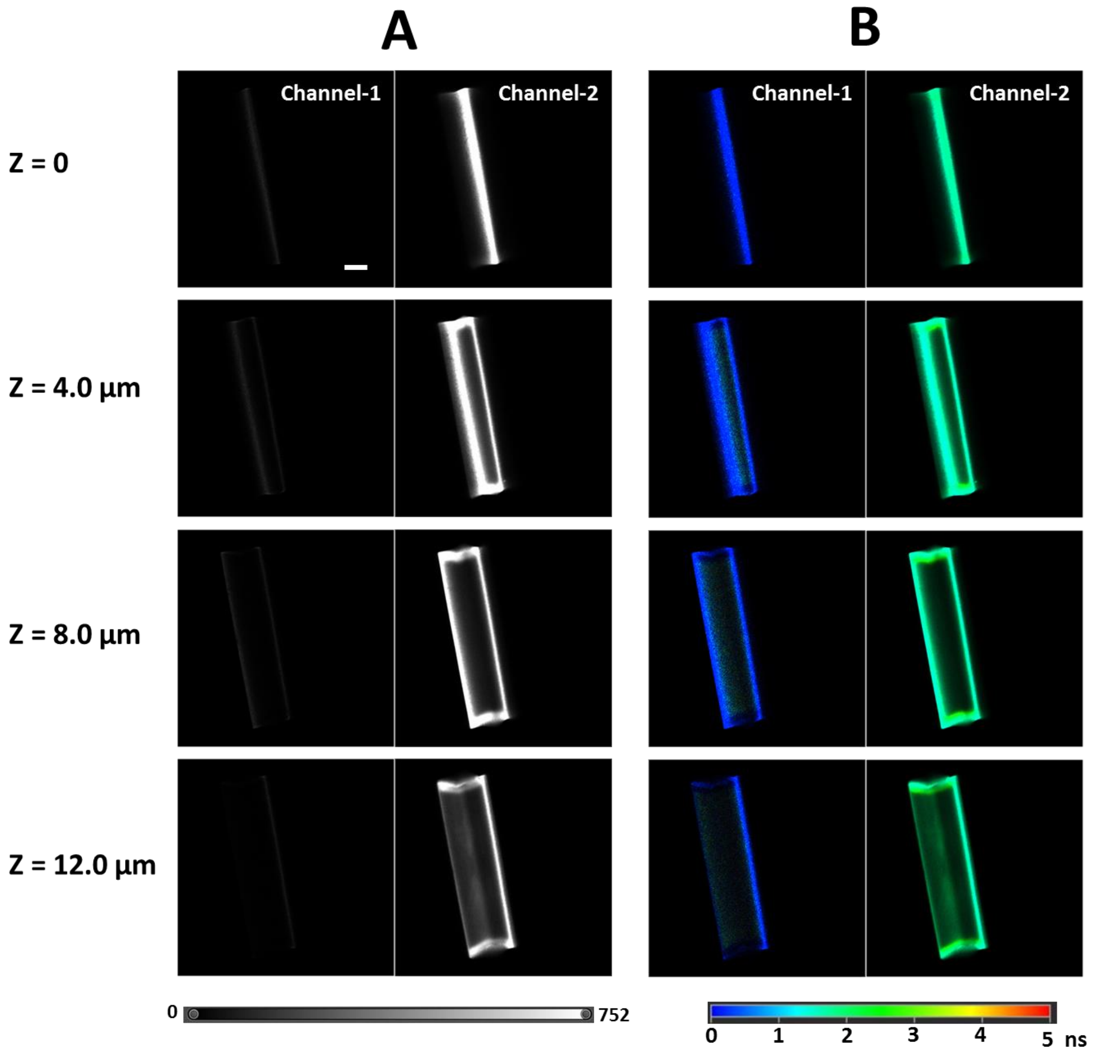

Figure S18. Z-scan images showing the fluorescence (A) intensity and (B) lifetime of crystals saturated with sodium fluorescein (**SF**, 0.03 mmol/g) and loaded with sodium resorufin (**SR3**, 4.9 × 10^{-3} mmol/g). Scale bar: 20 μm.

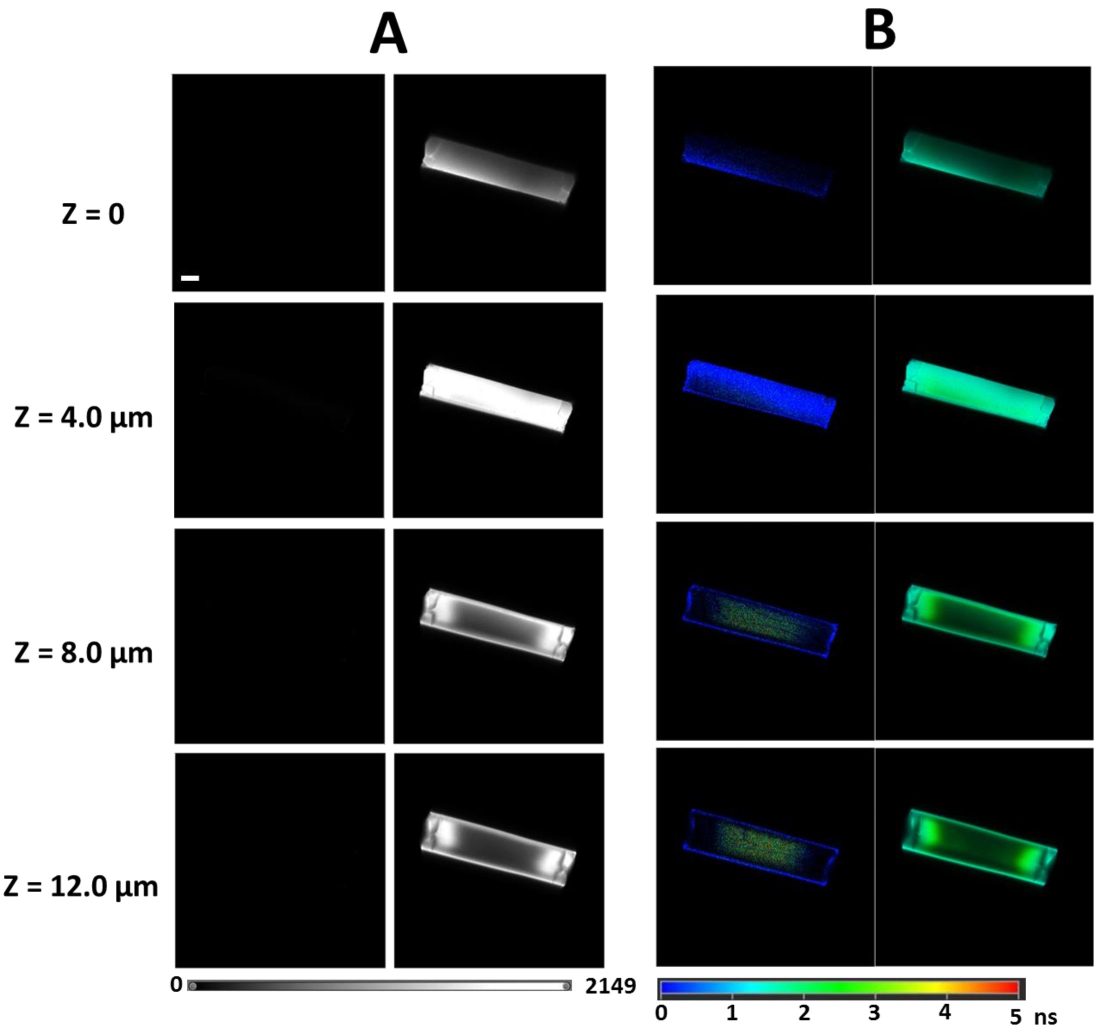

Figure S19. Z-scan images showing the fluorescence (A) intensity and (B) lifetime of crystals saturated with sodium fluorescein (**SF**, 0.03 mmol/g) and loaded with sodium resorufin (**SR4**, 4.1 × 10^{-2} mmol/g). Scale bar: 20 μm.

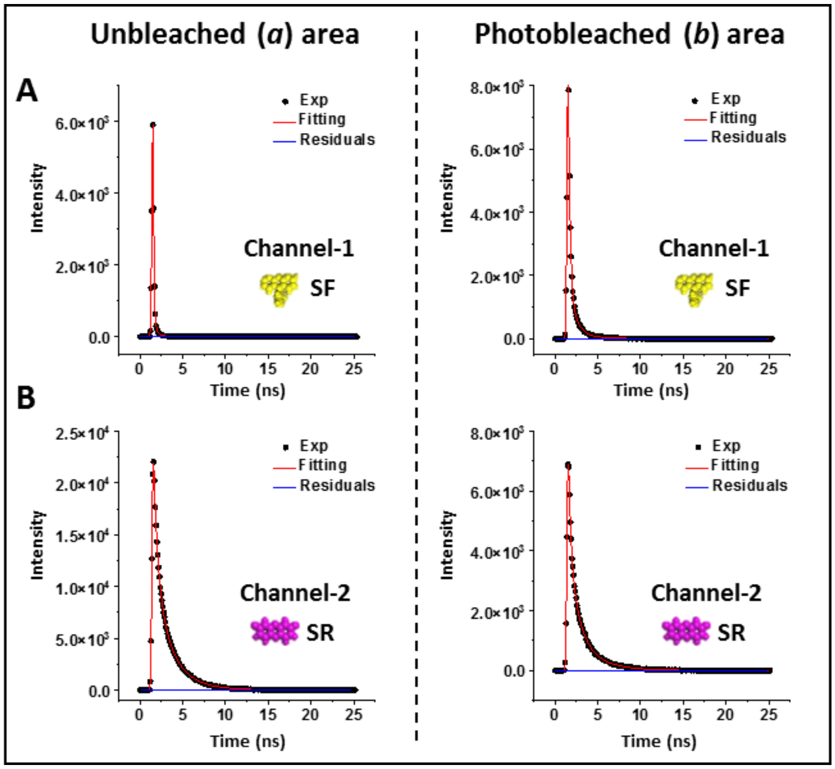

Figure S20. Fluorescence lifetimes of the bulk and curve fitting of experimental data shown in **Figure 7**.

Table S5. Lifetime analysis and curve fitting of experimental data shown in **Figure 7** and **Figure S13**. The fitted results are composed of three lifetime components (τ1, τ2, τ3). The average lifetime is also calculated including the intensity-weighted lifetime (τi) and amplitude-weighted lifetime (τa). Fluorescence lifetime imaging microscopy (FLIM) data analysis and fitting was performed using Leica application suite X (Leica Microsystems CNS GmbH) LAS X FLIM/FCS

	Unbleached	Photobleached	Unbleached	Photobleached
	**Channel-1 (SF) at "a" spot**	**Channel-1 (SF) at "b" spot**	**Channel-2 (SR) at "a" spot**	**Channel-2 (SR) at "b" spot**
Lifetime τ1 (ns)	0.089	0.226	0.429	0.255
Lifetime τ2 (ns)	0.378	0.987	1.514	1.069
Lifetime τ3 (ns)	1.978	3.668	2.819	2.784
Intensity weighted lifetime τi (ns)	0.238	0.772	1.793	1.703
Amplitude weighted lifetime τa (ns)	0.109	0.355	1.194	0.921
A1 (10^3)	9.989	7.953	13.001	4.561
A2 (10^3)	0.527	1.269	10.139	3.525
A3 (10^3)	0.030	0.067	4.116	1.352
I1 (10^3)	9.146	18.537	57.531	11.975
I2 (10^3)	2.054	12.917	158.269	38.873
I3 (10^3)	0.622	2.537	119.659	38.817
χ^2	0.978	1.129	0.978	0.823

References

[S1] M. Vasylyev, R. Popovitz-Biro, L. J. W. Shimon, R. Neumann, *J. Mol. Struct.* **2003**, *656*, 27–35.

[S2] L. R. Melby, R. J. Harder, W. R. Hertler, W. Mahler, R. E. Benson, W. E. Mochel, *J. Am. Chem. Soc.* **1962**, *84*, 3374–3387.

[S3] G. M. Sheldrick, *Acta Crystallogr. Sect. C* **2015**, *71*, 3–8.

[S4] A. L. Spek, *Acta Crystallogr. Sect. D* **2009**, *65*, 148–155.

[S5] O. V Dolomanov, L. J. Bourhis, R. J. Gildea, J. A. K. Howard, H. Puschmann, *J. Appl. Crystallogr.* **2009**, *42*, 339–341.
