# Supplementary material for: Energy Transport in Dichroic Metallo‐organic Crystals: Selective Inclusion of Spatially Resolved Arrays of Donor and Acceptor Dyes in Different Nanochannels
Source: Angew Chem Int Ed Engl. 2022 Dec 16;62(4):e202214041. doi: 10.1002/anie.202214041 (PMC10107947; doi:10.1002/anie.202214041)

checkCIF/PLATON report

Structure factors have been supplied for datablock(s) v375os

THIS REPORT IS FOR GUIDANCE ONLY. IF USED AS PART OF A REVIEW PROCEDURE FOR PUBLICATION, IT SHOULD NOT REPLACE THE EXPERTISE OF AN EXPERIENCED CRYSTALLOGRAPHIC REFEREE.

No syntax errors found. CIF dictionary Interpreting this report

Datablock: v375os

Bond precision:	C-C = 0.0106 A		Wavelength=1.54184
Cell:	a=26.3679(5)	b=26.3679(5)	c=18.1682(3)
	alpha=90	beta=90	gamma=120
Temperature:	100 K		

	Calculated	Reported
Volume	10939.4(5)	10939.4(5)
Space group	P 6 2 2	P 6 2 2
Hall group	P 6 2	P 6 2
Moiety formula	C62 H54 Cd Cl N4 O [+ solvent]	C62 H54 Cd Cl N4 O
Sum formula	C62 H54 Cd Cl N4 O [+ solvent]	C62 H54 Cd Cl N4 O
Mr	1018.95	1018.94
Dx,g cm-3	0.928	0.928
Z	6	6
Mu (mm-1)	2.981	2.981
F000	3162.0	3162.0
F000’	3172.43	
h,k,lmax	33,33,22	33,27,21
Nref	7781[4426]	7486
Tmin,Tmax	0.614,0.699	0.374,1.000
Tmin’	0.557	

Correction method= # Reported T Limits: Tmin=0.374 Tmax=1.000
AbsCorr = MULTI-SCAN

Data completeness= 1.69/0.96 Theta(max)= 77.246

R(reflections)= 0.0662(5458) wR2(reflections)= 0.2090(7486)

S = 1.040 Npar= 299

The following ALERTS were generated. Each ALERT has the format
test-name_ALERT_alert-type_alert-level.
Click on the hyperlinks for more details of the test.

Alert level B

PLAT420_ALERT_2_B D-H Bond Without Acceptor O1 --H1A . Please Check

Author Response: O1 is water coordinated to Cd metal center

PLAT420_ALERT_2_B D-H Bond Without Acceptor O1 --H1B . Please Check

Author Response: O1 is water coordinated to Cd metal center

Alert level C

```
PLAT234_ALERT_4_C Large Hirshfeld Difference C10      --C11     .        0.17 Ang.
PLAT234_ALERT_4_C Large Hirshfeld Difference C10      --C13     .        0.22 Ang.
PLAT234_ALERT_4_C Large Hirshfeld Difference C53      --C54     .        0.16 Ang.
PLAT234_ALERT_4_C Large Hirshfeld Difference C56      --C57_b   .        0.16 Ang.
PLAT234_ALERT_4_C Large Hirshfeld Difference C57      --C56_a   .        0.16 Ang.
PLAT241_ALERT_2_C High   'MainMol' Ueq as Compared to Neighbors of        C13 Check
PLAT241_ALERT_2_C High   'MainMol' Ueq as Compared to Neighbors of        C14 Check
PLAT241_ALERT_2_C High   'MainMol' Ueq as Compared to Neighbors of        C16 Check
PLAT241_ALERT_2_C High   'MainMol' Ueq as Compared to Neighbors of        C24 Check
PLAT241_ALERT_2_C High   'MainMol' Ueq as Compared to Neighbors of        C54 Check
PLAT241_ALERT_2_C High   'MainMol' Ueq as Compared to Neighbors of        C55 Check
PLAT242_ALERT_2_C Low    'MainMol' Ueq as Compared to Neighbors of         N1 Check
PLAT242_ALERT_2_C Low    'MainMol' Ueq as Compared to Neighbors of         N2 Check
PLAT242_ALERT_2_C Low    'MainMol' Ueq as Compared to Neighbors of        C15 Check
PLAT242_ALERT_2_C Low    'MainMol' Ueq as Compared to Neighbors of        C20 Check
PLAT242_ALERT_2_C Low    'MainMol' Ueq as Compared to Neighbors of        C23 Check
PLAT260_ALERT_2_C Large Average Ueq of Residue Including       Cd1      0.102 Check
PLAT342_ALERT_3_C Low Bond Precision on  C-C Bonds ...............    0.01065 Ang.
PLAT368_ALERT_2_C Short  C(sp2)-C(sp2) Bond  C56      - C57_b   .        1.22 Ang.
PLAT790_ALERT_4_C Centre of Gravity not Within Unit Cell: Resd.  #          1 Note
              C62 H54 Cd Cl N4 O
PLAT906_ALERT_3_C Large K Value in the Analysis of Variance ......      2.001 Check
PLAT911_ALERT_3_C Missing FCF Refl Between Thmin & STh/L=    0.600          4 Report
PLAT918_ALERT_3_C Reflection(s) with I(obs) much Smaller I(calc) .          1 Check
```

Alert level G

```
PLAT002_ALERT_2_G Number of Distance or Angle Restraints on AtSite          4 Note
PLAT003_ALERT_2_G Number of Uiso or Uij Restrained non-H Atoms ...          9 Report
PLAT007_ALERT_5_G Number of Unrefined Donor-H Atoms ..............          2 Report
PLAT072_ALERT_2_G SHELXL First  Parameter in WGHT  Unusually Large       0.14 Report
```

```
PLAT172_ALERT_4_G The CIF-Embedded .res File Contains DFIX Records          5 Report
PLAT173_ALERT_4_G The CIF-Embedded .res File Contains DANG Records          3 Report
PLAT187_ALERT_4_G The CIF-Embedded .res File Contains RIGU Records          1 Report
PLAT232_ALERT_2_G Hirshfeld Test Diff (M-X)  Cd1      --Cl1      .       14.0 s.u.
PLAT300_ALERT_4_G Atom Site Occupancy of Cl1        Constrained at        0.5 Check
PLAT300_ALERT_4_G Atom Site Occupancy of O1         Constrained at        0.5 Check
PLAT300_ALERT_4_G Atom Site Occupancy of H1A        Constrained at        0.5 Check
PLAT300_ALERT_4_G Atom Site Occupancy of H1B        Constrained at        0.5 Check
PLAT300_ALERT_4_G Atom Site Occupancy of H3A        Constrained at        0.5 Check
PLAT300_ALERT_4_G Atom Site Occupancy of H3B        Constrained at        0.5 Check
PLAT300_ALERT_4_G Atom Site Occupancy of H6A        Constrained at        0.5 Check
PLAT300_ALERT_4_G Atom Site Occupancy of H6B        Constrained at        0.5 Check
PLAT301_ALERT_3_G Main Residue  Disorder ..............(Resd  1  )         3% Note
PLAT367_ALERT_2_G Long?  C(sp?)-C(sp?) Bond  C1       - C3       .       1.53 Ang.
PLAT367_ALERT_2_G Long?  C(sp?)-C(sp?) Bond  C5       - C6       .       1.51 Ang.
PLAT410_ALERT_2_G Short Intra H...H Contact  H3A      ..H25      .       2.14 Ang.
                                                      x,y,z  =      1_555 Check
PLAT410_ALERT_2_G Short Intra H...H Contact  H3B      ..H25      .       2.14 Ang.
                                             -2-y,-2-x,-1-z  =     10_334 Check
PLAT415_ALERT_2_G Short Inter D-H..H-X       H1A      ..H16      .       2.07 Ang.
                                                x,-1+x-y,-z  =     12_545 Check
PLAT606_ALERT_4_G Solvent Accessible VOID(S) in Structure ........          ! Info
PLAT804_ALERT_5_G Number of ARU-Code Packing Problem(s) in PLATON          1 Info
PLAT860_ALERT_3_G Number of Least-Squares Restraints ............         58 Note
PLAT883_ALERT_1_G No Info/Value for _atom_sites_solution_primary .     Please Do !
PLAT912_ALERT_4_G Missing # of FCF Reflections Above STh/L=  0.600        69 Note
PLAT951_ALERT_5_G Calculated (ThMax) and CIF-Reported Kmax Differ          6 Units
PLAT961_ALERT_5_G Dataset Contains no Negative Intensities .......     Please Check
PLAT978_ALERT_2_G Number C-C Bonds with Positive Residual Density.          0 Info
```

---

  0 **ALERT level A** = Most likely a serious problem - resolve or explain
  2 **ALERT level B** = A potentially serious problem, consider carefully
 23 **ALERT level C** = Check. Ensure it is not caused by an omission or oversight
 30 **ALERT level G** = General information/check it is not something unexpected

  1 ALERT type 1 CIF construction/syntax error, inconsistent or missing data
 25 ALERT type 2 Indicator that the structure model may be wrong or deficient
  6 ALERT type 3 Indicator that the structure quality may be low
 19 ALERT type 4 Improvement, methodology, query or suggestion
  4 ALERT type 5 Informative message, check

It is advisable to attempt to resolve as many as possible of the alerts in all categories. Often the minor alerts point to easily fixed oversights, errors and omissions in your CIF or refinement strategy, so attention to these fine details can be worthwhile. In order to resolve some of the more serious problems it may be necessary to carry out additional measurements or structure refinements. However, the purpose of your study may justify the reported deviations and the more serious of these should normally be commented upon in the discussion or experimental section of a paper or in the "special_details" fields of the CIF. checkCIF was carefully designed to identify outliers and unusual parameters, but every test has its limitations and alerts that are not important in a particular case may appear. Conversely, the absence of alerts does not guarantee there are no aspects of the results needing attention. It is up to the individual to critically assess their own results and, if necessary, seek expert advice.

Publication of your CIF in IUCr journals

A basic structural check has been run on your CIF. These basic checks will be run on all CIFs submitted for publication in IUCr journals (*Acta Crystallographica*, *Journal of Applied Crystallography*, *Journal of Synchrotron Radiation*); however, if you intend to submit to *Acta Crystallographica Section C* or *E* or *IUCrData*, you should make sure that full publication checks are run on the final version of your CIF prior to submission.

Publication of your CIF in other journals

Please refer to the *Notes for Authors* of the relevant journal for any special instructions relating to CIF submission.

PLATON version of 12/09/2022; check.def file version of 09/08/2022

Datablock v375os - ellipsoid plot

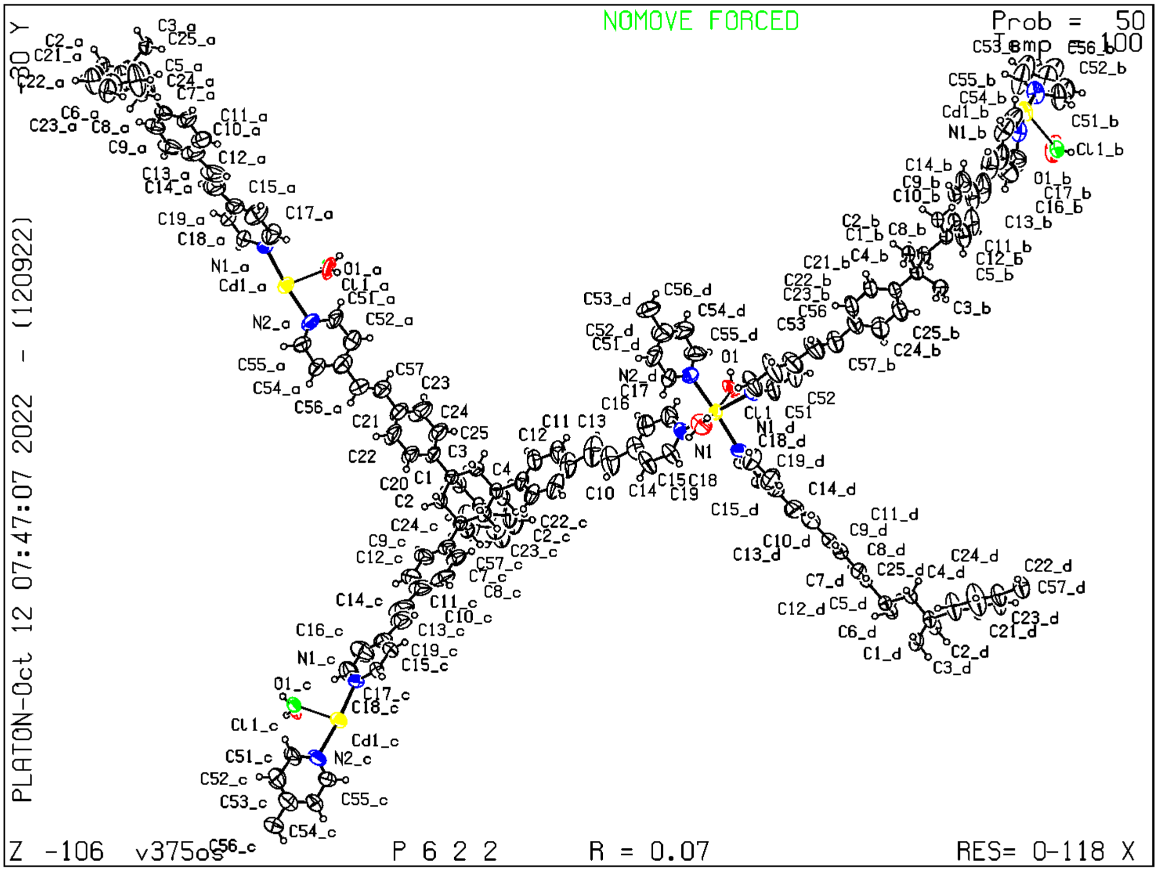

Supplement: Supplementary file 6 — Supporting Information [file ANIE-62-0-s002.pdf]
