# Supplementary material for: Energy Transport in Dichroic Metallo‐organic Crystals: Selective Inclusion of Spatially Resolved Arrays of Donor and Acceptor Dyes in Different Nanochannels
Source: Angew Chem Int Ed Engl. 2022 Dec 16;62(4):e202214041. doi: 10.1002/anie.202214041 (PMC10107947; doi:10.1002/anie.202214041)

checkCIF/PLATON report

Structure factors have been supplied for datablock(s) v370-sr

THIS REPORT IS FOR GUIDANCE ONLY. IF USED AS PART OF A REVIEW PROCEDURE FOR PUBLICATION, IT SHOULD NOT REPLACE THE EXPERTISE OF AN EXPERIENCED CRYSTALLOGRAPHIC REFEREE.

No syntax errors found.   CIF dictionary   Interpreting this report

Datablock: v370-sr

Bond precision: C-C = 0.0124 A   Wavelength=1.54184

Cell:	a=26.1666(8)	b=26.1666(8)	c=18.2494(5)
	alpha=90	beta=90	gamma=120
Temperature:	100 K		

	Calculated	Reported
Volume	10821.2(7)	10821.2(7)
Space group	P 6 2 2	P 6 2 2
Hall group	P 6 2	P 6 2
Moiety formula	2(C62 H56 Cd N4 O2), O [+ solvent]	2(C62 H56 Cd N4 O2), O
Sum formula	C124 H112 Cd2 N8 O5 [+ solvent]	C124 H112 Cd2 N8 O5
Mr	2019.04	2019.01
Dx,g cm-3	0.929	0.929
Z	3	3
Mu (mm-1)	2.693	2.693
F000	3144.0	3144.0
F000’	3152.63	
h,k,lmax	29,29,20	23,29,20
Nref	5688[3279]	5607
Tmin,Tmax	0.720,0.851	0.637,1.000
Tmin’	0.653	

Correction method= # Reported T Limits: Tmin=0.637 Tmax=1.000
AbsCorr = MULTI-SCAN

Data completeness= 1.71/0.99   Theta(max)= 61.863

R(reflections)= 0.0792(3749)   wR2(reflections)= 0.2706(5607)

S = 1.029   Npar= 286

The following ALERTS were generated. Each ALERT has the format
test-name_ALERT_alert-type_alert-level.
Click on the hyperlinks for more details of the test.

Alert level B

THETM01_ALERT_3_B The value of sine(theta_max)/wavelength is less than 0.575
Calculated sin(theta_max)/wavelength = 0.5719

Author Response: These are small weakly diffracting crystals with large voids Data were collected with Cu radiation at 100K to increase data resolution and improve quality.

PLAT241_ALERT_2_B High 'MainMol' Ueq as Compared to Neighbors of C15 Check

Author Response: the ligand has long arms that are quite flexible

PLAT242_ALERT_2_B Low 'MainMol' Ueq as Compared to Neighbors of C22 Check

Author Response: the ligand has long arms that are flexible

PLAT306_ALERT_2_B Isolated Oxygen Atom (H-atoms Missing ?) .... O2 Check

Author Response: yes, the oxygen from a solvent water, hydrogen atoms not placed.

PLAT306_ALERT_2_B Isolated Oxygen Atom (H-atoms Missing ?) .... O3 Check

Author Response: yes, the oxygen from a solvent water, hydrogen atoms not placed.

PLAT420_ALERT_2_B D-H Without Acceptor O1 --H1A . Please Check

Author Response: hydrogen atoms not placed

PLAT420_ALERT_2_B D-H Without Acceptor O1 --H1B . Please Check

Author Response: hydrogen atoms not placed

PLAT780_ALERT_1_B Coordinates do not Form a Properly Connected Set Please Do !

Author Response: due to disorder and refinement of parts

PLAT922_ALERT_1_B wR2 in the CIF and FCF Differ by .... 0.0059 Check

Author Response: Checked, I did not find the source of this slight difference

PLAT990_ALERT_1_B Deprecated .res/.hkl Input Style SQUEEZE Job ... ! Note

Author Response: Shelxl 2016/4 and 2013 were used.

Alert level C

```
PLAT018_ALERT_1_C _diffrn_measured_fraction_theta_max .NE. *_full          ! Check
PLAT084_ALERT_3_C High wR2 Value (i.e. > 0.25) ...................       0.27 Report
PLAT241_ALERT_2_C High   'MainMol' Ueq as Compared to Neighbors of         C8 Check
```

Author Response: the ligand has long arms that are quite flexible

```
PLAT241_ALERT_2_C High   'MainMol' Ueq as Compared to Neighbors of         C9 Check
```

Author Response: the ligand has long arms that are quite flexible

```
PLAT241_ALERT_2_C High   'MainMol' Ueq as Compared to Neighbors of        C18 Check
```

Author Response: the ligand has long arms that are quite flexible

```
PLAT241_ALERT_2_C High   'MainMol' Ueq as Compared to Neighbors of        C20 Check
```

Author Response: the ligand has long arms that are quite flexible

```
PLAT241_ALERT_2_C High   'MainMol' Ueq as Compared to Neighbors of        C21 Check
```

Author Response: the ligand has long arms that are quite flexible

```
PLAT241_ALERT_2_C High   'MainMol' Ueq as Compared to Neighbors of        C23 Check
```

Author Response: the ligand has long arms that are quite flexible

```
PLAT241_ALERT_2_C High   'MainMol' Ueq as Compared to Neighbors of        C25 Check
```

Author Response: the ligand has long arms that are quite flexible

```
PLAT241_ALERT_2_C High   'MainMol' Ueq as Compared to Neighbors of        C26 Check
```

Author Response: the ligand has long arms that are quite flexible

```
PLAT241_ALERT_2_C High   'MainMol' Ueq as Compared to Neighbors of        C30 Check
```

Author Response: the ligand has long arms that are quite flexible

```
PLAT242_ALERT_2_C Low    'MainMol' Ueq as Compared to Neighbors of         N2 Check
```

Author Response: the ligand has long arms that are flexible

```
PLAT242_ALERT_2_C Low    'MainMol' Ueq as Compared to Neighbors of         C1 Check
```

Author Response: the ligand has long arms that are flexible

```
PLAT242_ALERT_2_C Low    'MainMol' Ueq as Compared to Neighbors of        C17 Check
```

Author Response: the ligand has long arms that are flexible

```
PLAT242_ALERT_2_C Low    'MainMol' Ueq as Compared to Neighbors of        C19 Check
```

Author Response: the ligand has long arms that are flexible

```
PLAT242_ALERT_2_C Low    'MainMol' Ueq as Compared to Neighbors of        C27 Check
```

Author Response: the ligand has long arms that are flexible

```
PLAT250_ALERT_2_C Large U3/U1 Ratio for Average U(i,j) Tensor ....        2.3 Note
PLAT260_ALERT_2_C Large Average Ueq of Residue Including       Cd1      0.149 Check
PLAT260_ALERT_2_C Large Average Ueq of Residue Including        O2      0.182 Check
PLAT260_ALERT_2_C Large Average Ueq of Residue Including        O3      0.126 Check
PLAT342_ALERT_3_C Low Bond Precision on  C-C Bonds ...............    0.01239 Ang.
PLAT906_ALERT_3_C Large K Value in the Analysis of Variance ......      2.622 Check
PLAT911_ALERT_3_C Missing FCF Refl Between Thmin & STh/L=    0.572         23 Report
PLAT918_ALERT_3_C Reflection(s) with I(obs) much Smaller I(calc) .          1 Check
PLAT921_ALERT_1_C R1  in the CIF and FCF Differ by ..............     0.0013 Check
PLAT923_ALERT_1_C S    Values in the CIF and FCF Differ by .......      0.024 Check
PLAT975_ALERT_2_C Check Calcd Resid. Dens.  0.65A   From O3              0.41 eA-3
```

Alert level G

```
PLAT002_ALERT_2_G Number of Distance or Angle Restraints on AtSite         15 Note
PLAT003_ALERT_2_G Number of Uiso or Uij Restrained non-H Atoms ...         21 Report
PLAT004_ALERT_5_G Polymeric Structure Found with Maximum Dimension          3 Info
PLAT007_ALERT_5_G Number of Unrefined Donor-H Atoms ..............          2 Report
PLAT033_ALERT_4_G Flack x Value Deviates > 3.0 * sigma from Zero .      0.130 Note
PLAT072_ALERT_2_G SHELXL First  Parameter in WGHT  Unusually Large       0.20 Report
PLAT171_ALERT_4_G The CIF-Embedded .res File Contains EADP Records          1 Report
PLAT172_ALERT_4_G The CIF-Embedded .res File Contains DFIX Records         10 Report
PLAT173_ALERT_4_G The CIF-Embedded .res File Contains DANG Records          5 Report
PLAT186_ALERT_4_G The CIF-Embedded .res File Contains ISOR Records          1 Report
PLAT187_ALERT_4_G The CIF-Embedded .res File Contains RIGU Records          3 Report
PLAT300_ALERT_4_G Atom Site Occupancy of C12        Constrained at        0.5 Check
PLAT300_ALERT_4_G Atom Site Occupancy of C13        Constrained at        0.5 Check
PLAT300_ALERT_4_G Atom Site Occupancy of C33        Constrained at        0.5 Check
PLAT300_ALERT_4_G Atom Site Occupancy of C35        Constrained at        0.5 Check
PLAT300_ALERT_4_G Atom Site Occupancy of H4A        Constrained at        0.5 Check
PLAT300_ALERT_4_G Atom Site Occupancy of H4B        Constrained at        0.5 Check
PLAT300_ALERT_4_G Atom Site Occupancy of H12        Constrained at        0.5 Check
PLAT300_ALERT_4_G Atom Site Occupancy of H13        Constrained at        0.5 Check
PLAT300_ALERT_4_G Atom Site Occupancy of H33        Constrained at        0.5 Check
PLAT300_ALERT_4_G Atom Site Occupancy of H35        Constrained at        0.5 Check
PLAT300_ALERT_4_G Atom Site Occupancy of H99A       Constrained at        0.5 Check
PLAT300_ALERT_4_G Atom Site Occupancy of H99B       Constrained at        0.5 Check
PLAT301_ALERT_3_G Main Residue  Disorder .............(Resd  1  )         6% Note
PLAT335_ALERT_2_G Check Large C6 Ring C-C Range C9       -C33            0.16 Ang.
PLAT367_ALERT_2_G Long?  C(sp?)-C(sp?) Bond  C1       - C99      .       1.54 Ang.
```

```
PLAT367_ALERT_2_G Long?  C(sp?)-C(sp?) Bond  C3       - C4       .       1.51 Ang.
PLAT410_ALERT_2_G Short Intra H...H Contact  H8       ..H12      .       1.93 Ang.
                                                      x,y,z  =     1_555 Check
PLAT410_ALERT_2_G Short Intra H...H Contact  H10      ..H33      .       2.01 Ang.
                                                      x,y,z  =     1_555 Check
PLAT410_ALERT_2_G Short Intra H...H Contact  H13      ..H18      .       2.02 Ang.
                                                      x,y,z  =     1_555 Check
PLAT410_ALERT_2_G Short Intra H...H Contact  H15      ..H35      .       2.01 Ang.
                                                      x,y,z  =     1_555 Check
PLAT410_ALERT_2_G Short Intra H...H Contact  H20      ..H99A     .       2.12 Ang.
                                                      x,y,z  =     1_555 Check
PLAT410_ALERT_2_G Short Intra H...H Contact  H20      ..H99A     .       2.12 Ang.
                                                      x,y,z  =     1_555 Check
PLAT414_ALERT_2_G Short Intra D-H..H-X       H1B      ..H35              2.10 Ang.
                                                      x,y,z  =     1_555 Check
PLAT606_ALERT_4_G VERY LARGE Solvent Accessible VOID(S) in Structure        ! Info
PLAT764_ALERT_4_G Overcomplete CIF Bond List Detected (Rep/Expd) .       1.11 Ratio
PLAT794_ALERT_5_G Tentative Bond Valency for Cd1       (II)      .       1.75 Info
PLAT860_ALERT_3_G Number of Least-Squares Restraints .............        157 Note
PLAT869_ALERT_4_G ALERTS Related to the Use of SQUEEZE Suppressed           ! Info
PLAT883_ALERT_1_G No Info/Value for _atom_sites_solution_primary .     Please Do !
PLAT910_ALERT_3_G Missing # of FCF Reflection(s) Below Theta(Min).          1 Note
PLAT933_ALERT_2_G Number of OMIT Records in Embedded .res File ...          8 Note
PLAT950_ALERT_5_G Calculated (ThMax) and CIF-Reported Hmax Differ           6 Units
PLAT961_ALERT_5_G Dataset Contains no Negative Intensities .......     Please Check
PLAT978_ALERT_2_G Number C-C Bonds with Positive Residual Density.          1 Info
```

```
  0 ALERT level A = Most likely a serious problem - resolve or explain
 10 ALERT level B = A potentially serious problem, consider carefully
 27 ALERT level C = Check. Ensure it is not caused by an omission or oversight
 45 ALERT level G = General information/check it is not something unexpected

  7 ALERT type 1 CIF construction/syntax error, inconsistent or missing data
 40 ALERT type 2 Indicator that the structure model may be wrong or deficient
  9 ALERT type 3 Indicator that the structure quality may be low
 21 ALERT type 4 Improvement, methodology, query or suggestion
  5 ALERT type 5 Informative message, check
```

It is advisable to attempt to resolve as many as possible of the alerts in all categories. Often the minor alerts point to easily fixed oversights, errors and omissions in your CIF or refinement strategy, so attention to these fine details can be worthwhile. In order to resolve some of the more serious problems it may be necessary to carry out additional measurements or structure refinements. However, the purpose of your study may justify the reported deviations and the more serious of these should normally be commented upon in the discussion or experimental section of a paper or in the "special_details" fields of the CIF. checkCIF was carefully designed to identify outliers and unusual parameters, but every test has its limitations and alerts that are not important in a particular case may appear. Conversely, the absence of alerts does not guarantee there are no aspects of the results needing attention. It is up to the individual to critically assess their own results and, if necessary, seek expert advice.

Publication of your CIF in IUCr journals

A basic structural check has been run on your CIF. These basic checks will be run on all CIFs submitted for publication in IUCr journals (*Acta Crystallographica*, *Journal of Applied Crystallography*, *Journal of Synchrotron Radiation*); however, if you intend to submit to *Acta Crystallographica Section C* or *E* or *IUCrData*, you should make sure that full publication checks are run on the final version of your CIF prior to submission.

Publication of your CIF in other journals

Please refer to the *Notes for Authors* of the relevant journal for any special instructions relating to CIF submission.

PLATON version of 08/07/2020; check.def file version of 17/06/2020

Datablock v370-sr - ellipsoid plot

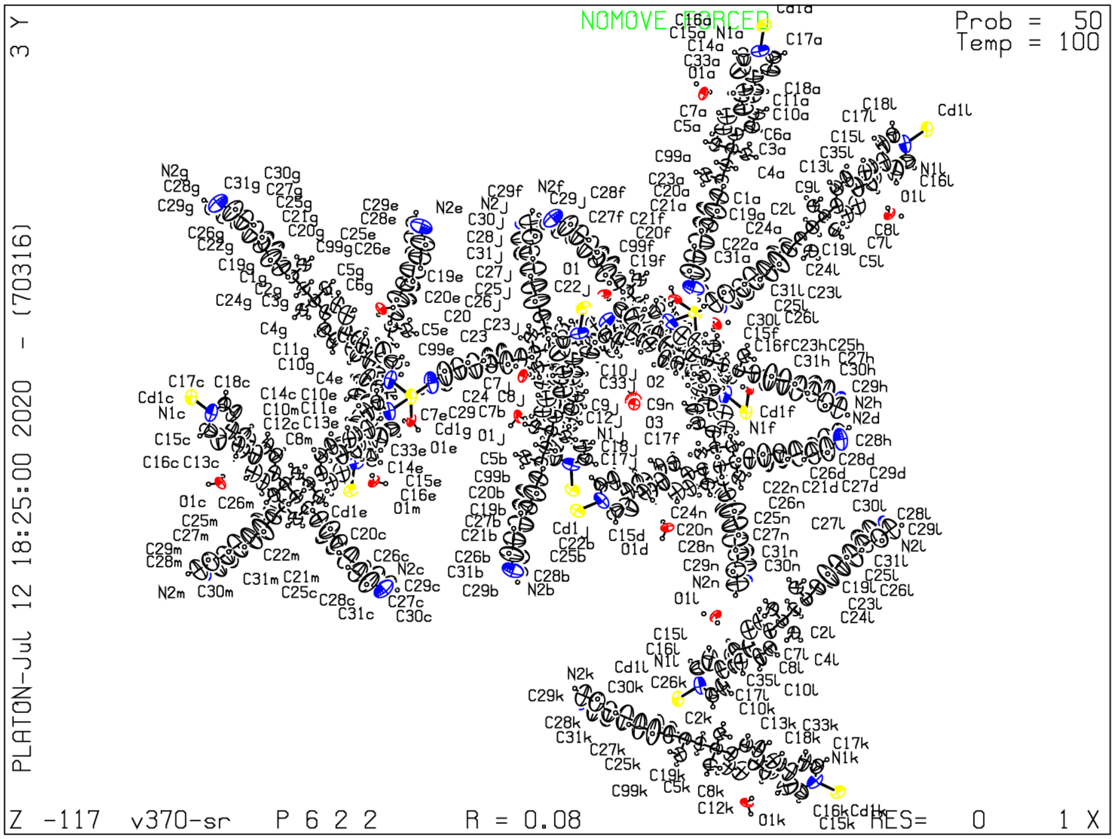

Supplement: Supplementary file 5 — Supporting Information [file ANIE-62-0-s003.pdf]
