# Supplementary material for: Energy Transport in Dichroic Metallo‐organic Crystals: Selective Inclusion of Spatially Resolved Arrays of Donor and Acceptor Dyes in Different Nanochannels
Source: Angew Chem Int Ed Engl. 2022 Dec 16;62(4):e202214041. doi: 10.1002/anie.202214041 (PMC10107947; doi:10.1002/anie.202214041)

checkCIF/PLATON report

Structure factors have been supplied for datablock(s) mr-v530

THIS REPORT IS FOR GUIDANCE ONLY. IF USED AS PART OF A REVIEW PROCEDURE FOR PUBLICATION, IT SHOULD NOT REPLACE THE EXPERTISE OF AN EXPERIENCED CRYSTALLOGRAPHIC REFEREE.

No syntax errors found. CIF dictionary Interpreting this report

Datablock: mr-v530

```
Bond precision:   C-C = 0.0222 A          Wavelength=1.54184

Cell:              a=26.4235(4)      b=26.4235(4)      c=18.0234(3)
                   alpha=90          beta=90           gamma=120
Temperature:       100 K
```

	Calculated	Reported
Volume	10898.0(4)	10898.0(4)
Space group	P 6 2 2	P 6 2 2
Hall group	P 6 2	P 6 2
Moiety formula	C62 H52 Cd Cl2 N4 [+ solvent]	C62 H52 Cd Cl2 N4
Sum formula	C62 H52 Cd Cl2 N4 [+ solvent]	C62 H52 Cd Cl2 N4
Mr	1036.39	1036.37
Dx,g cm-3	0.947	0.947
Z	6	6
Mu (mm-1)	3.319	3.319
F000	3204.0	3204.0
F000'	3216.37	
h,k,lmax	28,28,19	28,28,17
Nref	4772[2774]	4675
Tmin,Tmax	0.923,0.936	0.302,1.000
Tmin'	0.819	

```
Correction method= # Reported T Limits: Tmin=0.302 Tmax=1.000
AbsCorr = MULTI-SCAN

Data completeness= 1.69/0.98       Theta(max)= 55.990
```

R(reflections)= 0.0904(3533)   wR2(reflections)= 0.2896(4675)

S = 1.191   Npar= 265

The following ALERTS were generated. Each ALERT has the format
test-name_ALERT_alert-type_alert-level.
Click on the hyperlinks for more details of the test.

Alert level A

```
THETM01_ALERT_3_A  The value of sine(theta_max)/wavelength is less than 0.550
            Calculated sin(theta_max)/wavelength =    0.5376
```

Author Response: These were very small, weakly diffracting crystals. Data were collected on rotating anode Cu source to maximize resolution

---

Alert level B

```
PLAT094_ALERT_2_B Ratio of Maximum / Minimum Residual Density ....       4.51 Report
PLAT241_ALERT_2_B High   'MainMol' Ueq as Compared to Neighbors of        C12 Check
PLAT241_ALERT_2_B High   'MainMol' Ueq as Compared to Neighbors of        C20 Check
PLAT241_ALERT_2_B High   'MainMol' Ueq as Compared to Neighbors of        C31 Check
PLAT242_ALERT_2_B Low    'MainMol' Ueq as Compared to Neighbors of         N2 Check
PLAT242_ALERT_2_B Low    'MainMol' Ueq as Compared to Neighbors of        C19 Check
PLAT242_ALERT_2_B Low    'MainMol' Ueq as Compared to Neighbors of        C22 Check
PLAT342_ALERT_3_B Low Bond Precision on  C-C Bonds ...............    0.02218 Ang.
PLAT368_ALERT_2_B Short  C(sp2)-C(sp2) Bond  C12      - C26      .       1.13 Ang.
PLAT369_ALERT_2_B Long   C(sp2)-C(sp2) Bond  C14      - C35      .       1.57 Ang.
PLAT987_ALERT_1_B The Flack x is >> 0 -  Do a BASF/TWIN Refinement     Please Check
```

Alert level C

```
PLAT029_ALERT_3_C _diffrn_measured_fraction_theta_full value Low .      0.976 Why?
PLAT084_ALERT_3_C High wR2 Value (i.e. > 0.25) ...................       0.29 Report
PLAT213_ALERT_2_C Atom C12              has ADP max/min Ratio .....       3.7 prolat
PLAT213_ALERT_2_C Atom C26              has ADP max/min Ratio .....       3.7 prolat
PLAT213_ALERT_2_C Atom C27              has ADP max/min Ratio .....       3.3 prolat
PLAT213_ALERT_2_C Atom C31              has ADP max/min Ratio .....       3.7 prolat
PLAT220_ALERT_2_C NonSolvent   Resd 1   C   Ueq(max)/Ueq(min) Range       5.0 Ratio
PLAT222_ALERT_3_C NonSolvent Resd 1  H   Uiso(max)/Uiso(min) Range       4.6 Ratio
PLAT241_ALERT_2_C High   'MainMol' Ueq as Compared to Neighbors of         C9 Check
PLAT241_ALERT_2_C High   'MainMol' Ueq as Compared to Neighbors of        C16 Check
PLAT241_ALERT_2_C High   'MainMol' Ueq as Compared to Neighbors of        C24 Check
PLAT241_ALERT_2_C High   'MainMol' Ueq as Compared to Neighbors of        C28 Check
PLAT241_ALERT_2_C High   'MainMol' Ueq as Compared to Neighbors of        C30 Check
PLAT241_ALERT_2_C High   'MainMol' Ueq as Compared to Neighbors of        C35 Check
PLAT242_ALERT_2_C Low    'MainMol' Ueq as Compared to Neighbors of        Cd1 Check
PLAT242_ALERT_2_C Low    'MainMol' Ueq as Compared to Neighbors of         N1 Check
PLAT250_ALERT_2_C Large U3/U1 Ratio for Average U(i,j) Tensor ....        2.3 Note
PLAT260_ALERT_2_C Large Average Ueq of Residue Including       Cd1      0.215 Check
PLAT369_ALERT_2_C Long   C(sp2)-C(sp2) Bond  C9       - C33      .       1.55 Ang.
PLAT369_ALERT_2_C Long   C(sp2)-C(sp2) Bond  C26      - C27_e    .       1.53 Ang.
```

```
PLAT410_ALERT_2_C Short Intra H...H Contact  H2A      ..H24      .       1.95 Ang.
                                                      x,y,z  =      1_555 Check
PLAT911_ALERT_3_C Missing FCF Refl Between Thmin & STh/L=    0.538         20 Report
PLAT918_ALERT_3_C Reflection(s) with I(obs) much Smaller I(calc) .         17 Check
PLAT973_ALERT_2_C Check Calcd Positive Resid. Density on       Cd1       1.43 eA-3
```

Alert level G

```
PLAT002_ALERT_2_G Number of Distance or Angle Restraints on AtSite          7 Note
PLAT004_ALERT_5_G Polymeric Structure Found with Maximum Dimension          3 Info
PLAT033_ALERT_4_G Flack x Value Deviates > 3.0 * sigma from Zero .      0.075 Note
PLAT072_ALERT_2_G SHELXL First  Parameter in WGHT  Unusually Large       0.20 Report
PLAT171_ALERT_4_G The CIF-Embedded .res File Contains EADP Records          3 Report
PLAT172_ALERT_4_G The CIF-Embedded .res File Contains DFIX Records          4 Report
PLAT173_ALERT_4_G The CIF-Embedded .res File Contains DANG Records          3 Report
PLAT300_ALERT_4_G Atom Site Occupancy of H4A        Constrained at        0.5 Check
PLAT300_ALERT_4_G Atom Site Occupancy of H4B        Constrained at        0.5 Check
PLAT300_ALERT_4_G Atom Site Occupancy of H99A       Constrained at        0.5 Check
PLAT300_ALERT_4_G Atom Site Occupancy of H99B       Constrained at        0.5 Check
PLAT335_ALERT_2_G Check Large C6 Ring C-C Range C6       -C11            0.17 Ang.
PLAT367_ALERT_2_G Long?  C(sp?)-C(sp?) Bond  C3       - C4       .       1.52 Ang.
PLAT606_ALERT_4_G Solvent Accessible VOID(S) in Structure ........          ! Info
PLAT794_ALERT_5_G Tentative Bond Valency for Cd1       (II)      .       2.11 Info
PLAT860_ALERT_3_G Number of Least-Squares Restraints ............           7 Note
PLAT868_ALERT_4_G ALERTS Due to the Use of _smtbx_masks Suppressed          ! Info
PLAT883_ALERT_1_G No Info/Value for _atom_sites_solution_primary .     Please Do !
PLAT909_ALERT_3_G Percentage of I>2sig(I) Data at Theta(Max) Still        41% Note
PLAT910_ALERT_3_G Missing # of FCF Reflection(s) Below Theta(Min).          3 Note
PLAT933_ALERT_2_G Number of HKL-OMIT Records in Embedded .res File          6 Note
PLAT952_ALERT_5_G Calculated (ThMax) and CIF-Reported Lmax Differ.          2 Units
PLAT958_ALERT_1_G Calculated (ThMax) and Actual (FCF) Lmax Differ.          2 Units
PLAT978_ALERT_2_G Number C-C Bonds with Positive Residual Density.          1 Info
```

---

1 **ALERT level A** = Most likely a serious problem - resolve or explain
11 **ALERT level B** = A potentially serious problem, consider carefully
24 **ALERT level C** = Check. Ensure it is not caused by an omission or oversight
24 **ALERT level G** = General information/check it is not something unexpected

3 ALERT type 1 CIF construction/syntax error, inconsistent or missing data
34 ALERT type 2 Indicator that the structure model may be wrong or deficient
10 ALERT type 3 Indicator that the structure quality may be low
10 ALERT type 4 Improvement, methodology, query or suggestion
3 ALERT type 5 Informative message, check

It is advisable to attempt to resolve as many as possible of the alerts in all categories. Often the minor alerts point to easily fixed oversights, errors and omissions in your CIF or refinement strategy, so attention to these fine details can be worthwhile. In order to resolve some of the more serious problems it may be necessary to carry out additional measurements or structure refinements. However, the purpose of your study may justify the reported deviations and the more serious of these should normally be commented upon in the discussion or experimental section of a paper or in the "special_details" fields of the CIF. checkCIF was carefully designed to identify outliers and unusual parameters, but every test has its limitations and alerts that are not important in a particular case may appear. Conversely, the absence of alerts does not guarantee there are no aspects of the results needing attention. It is up to the individual to critically assess their own results and, if necessary, seek expert advice.

Publication of your CIF in IUCr journals

A basic structural check has been run on your CIF. These basic checks will be run on all CIFs submitted for publication in IUCr journals (*Acta Crystallographica*, *Journal of Applied Crystallography*, *Journal of Synchrotron Radiation*); however, if you intend to submit to *Acta Crystallographica Section C* or *E* or *IUCrData*, you should make sure that full publication checks are run on the final version of your CIF prior to submission.

Publication of your CIF in other journals

Please refer to the *Notes for Authors* of the relevant journal for any special instructions relating to CIF submission.

PLATON version of 18/05/2022; check.def file version of 17/05/2022

Datablock mr-v530 - ellipsoid plot

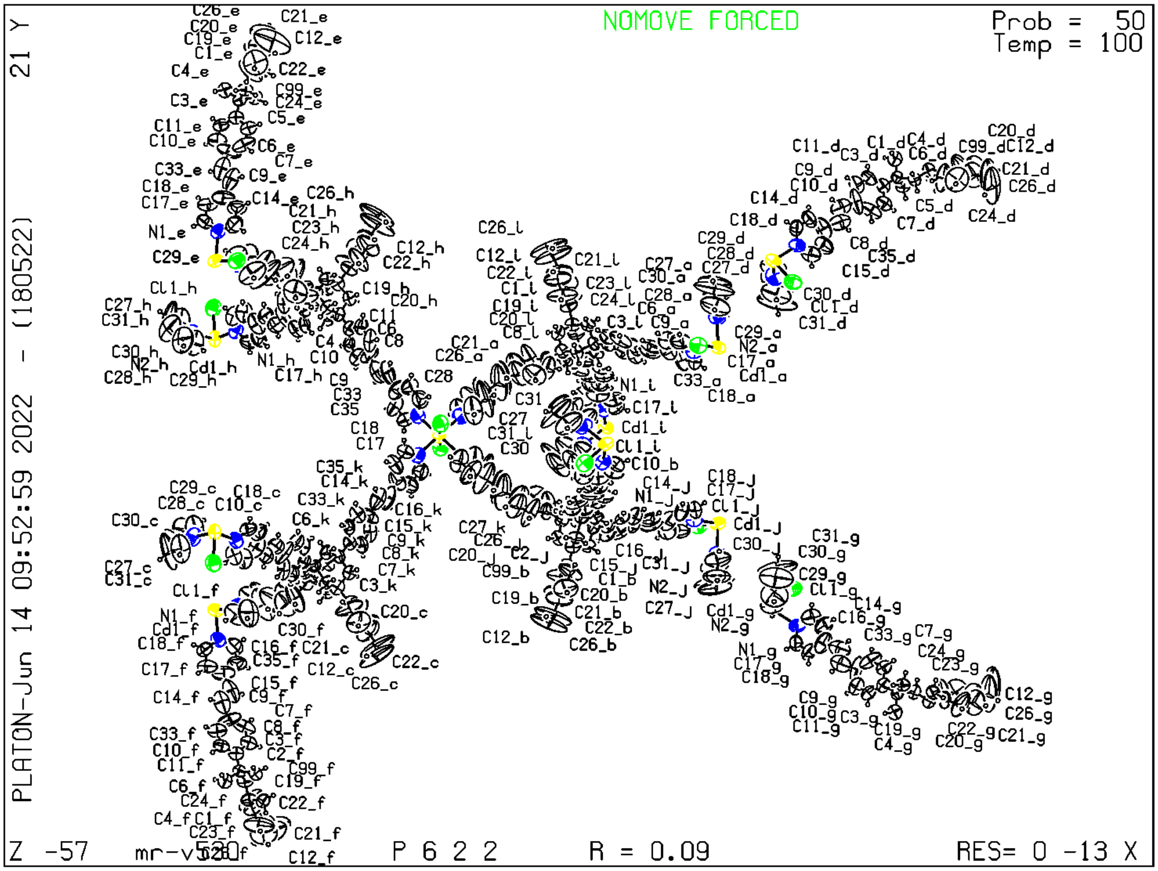

Supplement: Supplementary file 7 — Supporting Information [file ANIE-62-0-s005.pdf]
